# Dynamic Contacts of U2, RES, Cwc25, Prp8 and Prp45 Proteins with the Pre-mRNA Branch-Site and 3' Splice Site during Catalytic Activation and Step 1 Catalysis in Yeast Spliceosomes

**DOI:** 10.1371/journal.pgen.1005539

**Published:** 2015-09-22

**Authors:** Cornelius Schneider, Dmitry E. Agafonov, Jana Schmitzová, Klaus Hartmuth, Patrizia Fabrizio, Reinhard Lührmann

**Affiliations:** Max Planck Institute for Biophysical Chemistry, Department of Cellular Biochemistry, Göttingen, Germany; University of Chicago, UNITED STATES

## Abstract

Little is known about contacts in the spliceosome between proteins and intron nucleotides surrounding the pre-mRNA branch-site and their dynamics during splicing. We investigated protein-pre-mRNA interactions by UV-induced crosslinking of purified yeast B^act^ spliceosomes formed on site-specifically labeled pre-mRNA, and analyzed their changes after conversion to catalytically-activated B* and step 1 C complexes, using a purified splicing system. Contacts between nucleotides upstream and downstream of the branch-site and the U2 SF3a/b proteins Prp9, Prp11, Hsh49, Cus1 and Hsh155 were detected, demonstrating that these interactions are evolutionarily conserved. The RES proteins Pml1 and Bud13 were shown to contact the intron downstream of the branch-site. A comparison of the B^act^ crosslinking pattern versus that of B* and C complexes revealed that U2 and RES protein interactions with the intron are dynamic. Upon step 1 catalysis, Cwc25 contacts with the branch-site region, and enhanced crosslinks of Prp8 and Prp45 with nucleotides surrounding the branch-site were observed. Cwc25’s step 1 promoting activity was not dependent on its interaction with pre-mRNA, indicating it acts via protein-protein interactions. These studies provide important insights into the spliceosome's protein-pre-mRNA network and reveal novel RNP remodeling events during the catalytic activation of the spliceosome and step 1 of splicing.

## Author Summary

The spliceosome is a highly dynamic RNP machine that during the catalytic cycle undergoes many changes in composition and conformation. The pre-catalytic B^act^ spliceosome contains the U2, U6 and U5 snRNAs and ~40 proteins, which are evolutionarily conserved between budding yeast and metazoans. The B^act^ spliceosome is converted to a catalytically-activated B* spliceosome and following recruitment of the Cwc25 protein, step 1 of splicing is catalyzed and the C spliceosome is generated. The U2 snRNP plays an essential role in branch-site selection and pre-mRNA splicing catalysis. During the B^act^ to B* transition the affinity of several U2 SF3a/b proteins for the spliceosome is significantly reduced. Whether this is due to remodeling events affecting U2 snRNP contacts with the pre-mRNA is not known. Information about conserved spliceosomal protein-pre-mRNA contacts and their dynamics during splicing remains limited. Here we investigated pre-mRNA–protein contact sites in yeast B^act^ spliceosomes by UV-induced crosslinking. We detected contacts of nucleotides surrounding the branch-site with several of the U2 SF3a/b proteins, and we show that these interactions are evolutionarily conserved. We carried out a similar investigation with B* and C spliceosomes and provide important insights into the dynamics of pre-mRNA–protein interactions involving the essential U2, RES, Cwc25, Prp8 and Prp45 proteins.

## Introduction

The removal of introns from nuclear pre-mRNAs proceeds by way of two phosphoester transfer reactions and is catalyzed by the spliceosome, a large ribonucleoprotein (RNP) complex composed of the snRNPs U1, U2, U4/U6 and U5 and several proteins [1]. The spliceosome is a highly dynamic RNP machine that undergoes many changes in composition and conformation during its work cycle [2].

Initially, the U1 snRNP recognizes the 5’ splice site (5’ SS) and U2 snRNP recognizes the branch-site (BS) of the pre-mRNA, resulting in the formation of the pre-spliceosome or A complex. The pre-formed U4/U6.U5 tri-snRNP is then recruited, generating the B complex, which does not yet have an active site. Subsequent activation of the spliceosome (leading to the B^act^ complex) involves major rearrangement of the spliceosomal RNA–RNA interaction network. This rearrangement is catalyzed by the combined action of the RNA helicases Prp28 and Brr2 and leads to the displacement of the U1 and U4 snRNAs and the formation of new base-pair interactions between the U2 and U6 snRNAs and the 5’ SS [3]. Thus, a web of RNA–RNA interactions holds the 5’ SS and the BS together for step 1 catalysis [4].

The B^act^ complex, which contains U2, U6 and U5 and ~40 proteins in the yeast *S*. *cerevisiae* [5], is converted by the DEAH-box NTPase Prp2, in co-operation with the G-patch protein Spp2, into a catalytically activated complex (B*) [6–8]. Following the recruitment of the splicing factor Cwc25 [7,9], step 1 is catalyzed, whereby the 2’-OH of the BS adenosine attacks the 5' SS of the pre-mRNA generating the cleaved 5’ exon and intron 3’ exon; concomitantly the C complex is formed. This then catalyses step 2, in which the 3’ SS is cleaved, resulting in the excision of the intron and ligation of the 5’ and 3’ exons, after which the mRNA product is released. The excised intron lariat remains associated with U2, U5 and U6 snRNPs, which then dissociate and take part in subsequent rounds of splicing.

During the transformation of complex B into B^act^, not only is the spliceosome's RNA network radically rearranged, but also its protein composition changes significantly; as a result, several proteins are released, while twelve B^act^ proteins are recruited. At the same time the NTC (nineteen complex) and the NTC-related proteins are stably integrated into the B^act^ complex [5]. Likewise, the three proteins comprising the RES (pre-mRNA retention and splicing) complex [10] are also stably integrated into the B^act^ complex.

Despite the substantial restructuring that the spliceosome has undergone at this point, it does not yet have a functional active site. Previous studies showed that the binding affinity of several proteins is significantly changed during the Prp2-mediated transition of B^act^ spliceosomes to catalytically activated B* spliceosomes [7,11]. During this step, the essential splicing factor Cwc24 is quantitatively displaced from the B* complex. The U2-associated SF3a and SF3b proteins Prp11 and Cus1 and the RES protein Bud13 all remain bound to the B* spliceosome under near-physiological conditions, but their binding is reduced at high salt concentrations [11]. The destabilization of these proteins' binding by Prp2 and Spp2 indicates that the structure of the catalytic core of the spliceosome near the BS is remodeled. This could lead to a proper 5’SS and BS configuration for nucleophilic attack on the 5' SS phosphodiester bond during step 1 catalysis [7,12]. However, while it is clear that the affinity of the U2 and RES proteins for the spliceosome is significantly reduced during catalytic activation, it is not known whether this implies remodeling events involving contact of U2 and RES proteins with the pre-mRNA. Likewise, information about the set of spliceosomal protein-pre-mRNA contacts and their dynamics during splicing remains limited, but is crucial for unraveling potential functions of spliceosomal proteins for the formation and maintenance of the spliceosome's RNA–RNA network during catalysis.

The U2 snRNA/BS interaction is established in the A complex and is thought to make the BS adenosine bulge out for nucleophilic attack on the 5’ SS during step 1 catalysis [13]. In human pre-spliceosomes and spliceosomes the U2 SF3a/b proteins help to recruit the U2 snRNP to the BS, and all of them except SF3b130 can be crosslinked, in a sequence-independent manner, to a region upstream of the BS (the so-called “anchoring site”), to the BS itself and to a region downstream of it [14,15]. The BS sequence is highly conserved in yeast but only weakly conserved in metazoans. Given the short length of the BS sequence, and its degeneracy in metazoans, it has been suggested that spliceosomal proteins function together with the U2 snRNA/BS duplex to tether the U2 snRNP to pre-mRNA during spliceosome assembly. In yeast there is perfect complementarity between the BS sequence and the U2 RNA. Thus the anchoring/stabilization of the U2 snRNP to the BS sequence in yeast could be different from that in human, and it may not depend critically on protein–RNA interactions. Although most of the U2 proteins in the yeast *S*. *cerevisiae* are evolutionarily conserved [16–18], it is not known whether they interact in a similar way with the BS region in yeast spliceosomes, or whether a site equivalent to the human anchoring site exists in yeast pre-mRNAs. So far, only Hsh155, the yeast homologue of human SF3b155, has been shown to crosslink to pre-mRNA between the BS and the 3'SS [19]. Furthermore, it was recently shown that the RES subunit Snu17 is in contact with the pre-mRNA downstream of the BS in proximity of U2-Hsh155 [20]. However, is it currently not known whether additional components of the yeast U2 SF3a/b and RES subunits make direct contact with the pre-mRNA in spliceosomal complexes.

To address these questions, we have investigated protein–pre-mRNA interactions by UV-induced crosslinking of purified yeast spliceosomes stalled at the B^act^ assembly stage or after conversion of B^act^ to B* and C complexes, using a purified yeast splicing system [7]. Results of these studies revealed contacts in B^act^ complexes between pre-mRNA nucleotides directly upstream of the BS and the yeast U2 proteins Prp9, Prp11, Hsh49, Cus1 and Hsh155; the latter were also in contact with the intron further downstream of the BS. Thus, these interactions are evolutionarily conserved between yeast and man. Consistent with previous results demonstrating a Snu17-pre-mRNA crosslink [20], we observed that also the RES components Pml1 and Bud13 crosslinked to the intron downstream of the BS in B^act^ complexes. Subsequent UV crosslinking with purified spliceosomes that had been stalled after catalytic activation by Prp2/Spp2 and consecutive step 1 catalysis by Cwc25 revealed remodeling events involving contacts between U2 SF3a/b proteins upstream of the BS and the RES proteins downstream of it. Finally, concomitantly with these remodeling events, enhanced contacts of Cwc25, Prp8 and the NTC-related protein Prp45 with the BS and/or 3'SS regions were observed. These studies thus provide novel insights into the extensive protein–pre-mRNA interactions and their dynamics within and surrounding the pre-mRNA BS and 3'SS regions during step 1 of splicing in yeast spliceosomes.

## Results

### UV crosslinking of affinity-purified B^act^ spliceosomes and identification of protein–pre-mRNA crosslinks by 2D gel electrophoresis

**Fig 1:**
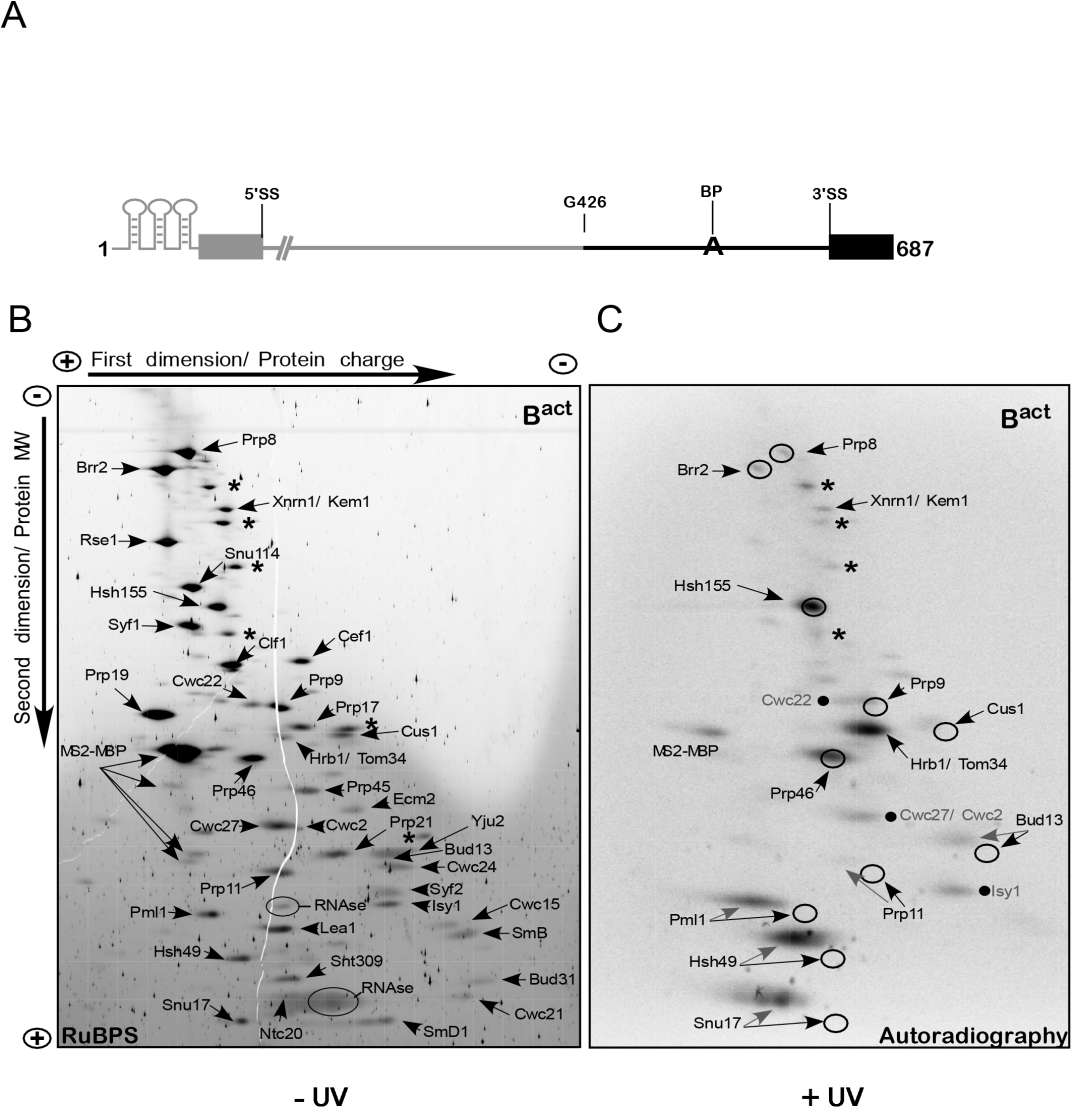
2D gel electrophoresis of affinity-purified yeast spliceosomal B^act ΔPrp2^ complexes. (A) Schematic representation of the 3’-region-labeled actin pre-mRNA which was used to assemble spliceosomal complexes for 2D gel electrophoresis. The 3’ portion of the pre-mRNA body-labeled with ^32^P-UTP is shown in black and includes the guanosine at position 426 of the intron up to the end of the 3’ exon. The unlabeled pre-mRNA is shown in gray. (B) B^act^ complexes were purified according to protocol 1 (S1 Text). Total proteins of purified, non-irradiated (–UV) and RNase-digested B^act^ complexes were separated electrophoretically by 2D gel electrophoresis and then stained with RuBPS [23]. The directions of the first-dimension and second-dimension electrophoreses are shown at the top and on the left. In the first-dimension of gel electrophoresis, proteins are separated by charge while in the second-dimension they are separated according to their molecular weight. The proteins observed in the predominant spots were cut from the gel and analyzed by mass spectrometry, and the proteins identified are indicated. Two predominant spots corresponding to the contaminant proteins Xrn1 and Hrb1 are indicated; additional predominant spots corresponding to contaminant proteins are labeled with asterisks, from top to bottom: Prp5, Scp160, Kre33, Sup35, Bfr1 and Nop1. Those corresponding to RNases and MS2-MBP are also indicated. (C) Autoradiography of the 2D gel comprising total proteins of purified, UV-irradiated (+UV) and RNase digested B^act^ complexes. The circles indicate the position of the RuBPS stained spots shown in (B). Radioactive spots corresponding to proteins that were not further characterized in this work are indicated by a dot, from top to bottom: Cwc22, Cwc2/Cwc27, Isy1.

To obtain insights into the nature and number of proteins that are in direct contact with the region at the 3’ end of the intron in purified yeast spliceosomes, we constructed a pre-mRNA which was body-labeled with ^32^P-UTP during transcription in the 3’ third of the intron, including exon 2 and 47 nucleotides (nts) upstream of the BS (termed hereinafter “3’-region-labeled pre-mRNA”; Fig 1A). The experimental strategy used to produce the 3’-region-labeled pre-mRNA is outlined in S1A Fig. Briefly, the 3’ fragment was obtained by T7 transcription. For this purpose, a T7 promoter was added by PCR and the PCR product was transcribed in vitro with an excess of GMP to ensure the presence of a monophosphate at the 5’ end and with α-^32^P UTP to randomly trace-label the entire RNA transcript (see S1 Text for details). To produce the 5’ fragment, unlabeled actin pre-mRNA, prepared by transcription in vitro, was specifically cleaved between nucleotides 425 and 426 by a DNA enzyme based on the “8–17” catalytic motif [21] (S1A Fig, upper panel). The 5’ cleavage fragment was dephosphorylated, gel purified and ligated to the T7-transcribed 3’ fragment by DNA splint directed RNA ligation [22]. The 3’-region-labeled pre-mRNA allows the analysis by UV crosslinking of protein–pre-mRNA interactions at the BS site, the region directly upstream of the BS as well as around the 3’SS.

Protein–pre-mRNA interactions were analyzed initially in purified B^act^ spliceosomes, which were assembled *in vitro* by incubating heat-inactivated splicing extracts from a temperature-sensitive *prp2*-1 yeast strain with the 3’-region-labeled pre-mRNA that also contained an MS2 binding site at its 5’ end [5,7]. B^act^ spliceosomes were purified by glycerol-gradient centrifugation and MS2-MBP affinity chromatography and then were irradiated with UV light at 254 nm, and digested under denaturing conditions with a mixture of RNases T1, A and I. The entire protein mixture was then separated by two-dimensional (2D) gel electrophoresis as described for human spliceosomal complexes [23]. Our 2D gel electrophoresis method is based on charge-driven separation of proteins under denaturing conditions at acidic pH in the first dimension and further separated by molecular weight though SDS gradient PAGE in the second. In contrast to the commonly used isoelectric focusing (IEF), this system prevents proteins from reaching zero charge and allows separation without in-gel precipitation over a wide range of isoelectric points (IEPs) and with masses greater than 300 kDa [23].

Fig 1B shows a RuBPS-stained 2D gel (S1 Text) of the total proteins isolated from non-irradiated B^act^ complexes. Individual protein spots were cut out of the gel and peptides were identified by mass spectrometry (MS). Only a few contaminant proteins were found, such as Xnrn1/Kem1 and Hrb1/Tom34, which are usually present in small amounts in preparations of yeast spliceosomes [5,7]. All the previously identified B^act^ complex proteins were observed [5]; these included nearly all of the U2 SF3a/b proteins (i.e. SF3b: Rse1, Hsh155, Cus1 and Hsh49; SF3a: Prp9, Prp11 and Prp21), which could be well separated from each other. The low-MW U2 proteins Msl1, Rds3 and Ysf3 and the Sm proteins D2, E, F and G ran out of the gel but could be identified in 2D gels which were modified to improve the resolution of smaller proteins [23]. A subset of U5 proteins (Prp8, Brr2 and Snu114), and most proteins of the NTC complex and NTC-related proteins, were also located as single, distinct spots. Proteins of the RES complex (Ist3/Snu17, Pml1 and Bud13) [10] were also identified. A comparison of previous MS analyses of purified yeast B^act^ spliceosomal complexes [5,7,24] with those of our 2D analysis indicates a general reliability of this method for separating and identifying proteins that co-purify with yeast spliceosomal complexes [23].

Fig 1C shows an autoradiography of the 2D gel performed as described above but with UV-irradiated B^act^ complexes. Exposure to 254-nm UV light is known to induce direct (zero-length) crosslinks between nitrogenous bases of nucleic acids and amino-acid side chains when they are in a favorable configuration. We observed prominent ^32^P-labeled spots of U2-Hsh155 and the NTC-related protein Prp46, both of which could be superimposed on the RuBPS-stained spots (indicated by circles in Fig 1C). This indicates that the covalent attachment of a few RNA nts to proteins larger than 50 kDa, after UV-irradiation, did not alter significantly their migration behavior. A predominant crosslink in the middle of the autoradiogram was due to the contaminating poly(A)-binding protein Hrb1/Tom34, while other contaminant proteins were crosslinked to pre-mRNA at very low levels or not at all (marked by asterisks in Fig 1C). Prominent radioactive spots were also observed for smaller U2 proteins (MW < 50 kDa), such as U2-Hsh49 and also two proteins of the RES complex (Pml1 and Snu17). The covalent attachment of RNA nts to smaller proteins led to a shift of their crosslinked species to the acidic region (i.e. left side) of the gel in the first dimension; in addition, they were separated into several spots and did not co-localize with the RuBPS stained spot, which were located slightly below (Fig 1C). Nevertheless, in all three cases crosslinked species migrated to the left side of the gel, where no other co-migrating proteins were visible in the RuBPS stained gel, indicating that the crosslinked proteins of interest were not contaminated with other proteins. We recently showed that the RES complex subunit Snu17 crosslinks in the B^act^ complex to a 14-nt-long region of the pre-mRNA intron downstream of the BS, as shown after digestion with RNase T1 [20]. Thus, the presence of several spots in the 2D gel may indicate that crosslinked pre-mRNA–protein species included shorter digestion products of the 14-nt-long RNA fragment (note that treatment with three different RNases was performed for 2D gel analysis). The same is likely to be true for RES-Pml1 and U2-Hsh49, whose crosslinked species showed a similar separation behavior (Fig 1C). The unequivocal identification of these proteins will be demonstrated below.

We also observed that low levels of additional proteins crosslinked to the 3’ part of the intron, such as the U5 proteins Prp8 and Brr2, the U2 proteins Prp9, Prp11, Cus1, the RES protein Bud13 and a few B^act^-specific proteins (i.e. Cwc22, Cwc27/Cwc2 and Isy1, indicated by dots; Fig 1C). These proteins crosslinked much less strongly to the 3’ part of the intron in the B^act^ spliceosome than those described above, suggesting that they are in contact with the 3’ region of the pre-mRNA but are in a conformation that does not favor the formation of UV-induced crosslinks. Alternatively, digestion with a mixture of three different RNases before 2D gel electrophoresis may lead to partial digestion of the crosslinking site.

### Identification of crosslinks between U2 and RES proteins and defined regions of the intron within B^act^ spliceosomes

Next, we focused on the characterization of the crosslinked U2 SF3a/b and RES complex proteins with two major objectives: (i) identification of the candidate proteins crosslinked to pre-mRNA by pull-down of their tagged version, and (ii) identification of the region/position of crosslinks within the intron. Therefore, we generated yeast strains carrying a C-terminally TAP-tagged version of most of the U2 SF3a/b proteins and the two RES subunits Pml1 and Bud13. In addition, to localize protein–pre-mRNA interactions to well-defined short regions in the RNA stretch directly upstream of the BS or around and downstream of it, we synthesized site-specifically labeled pre-mRNA (S1B Fig).

**Fig 2:**
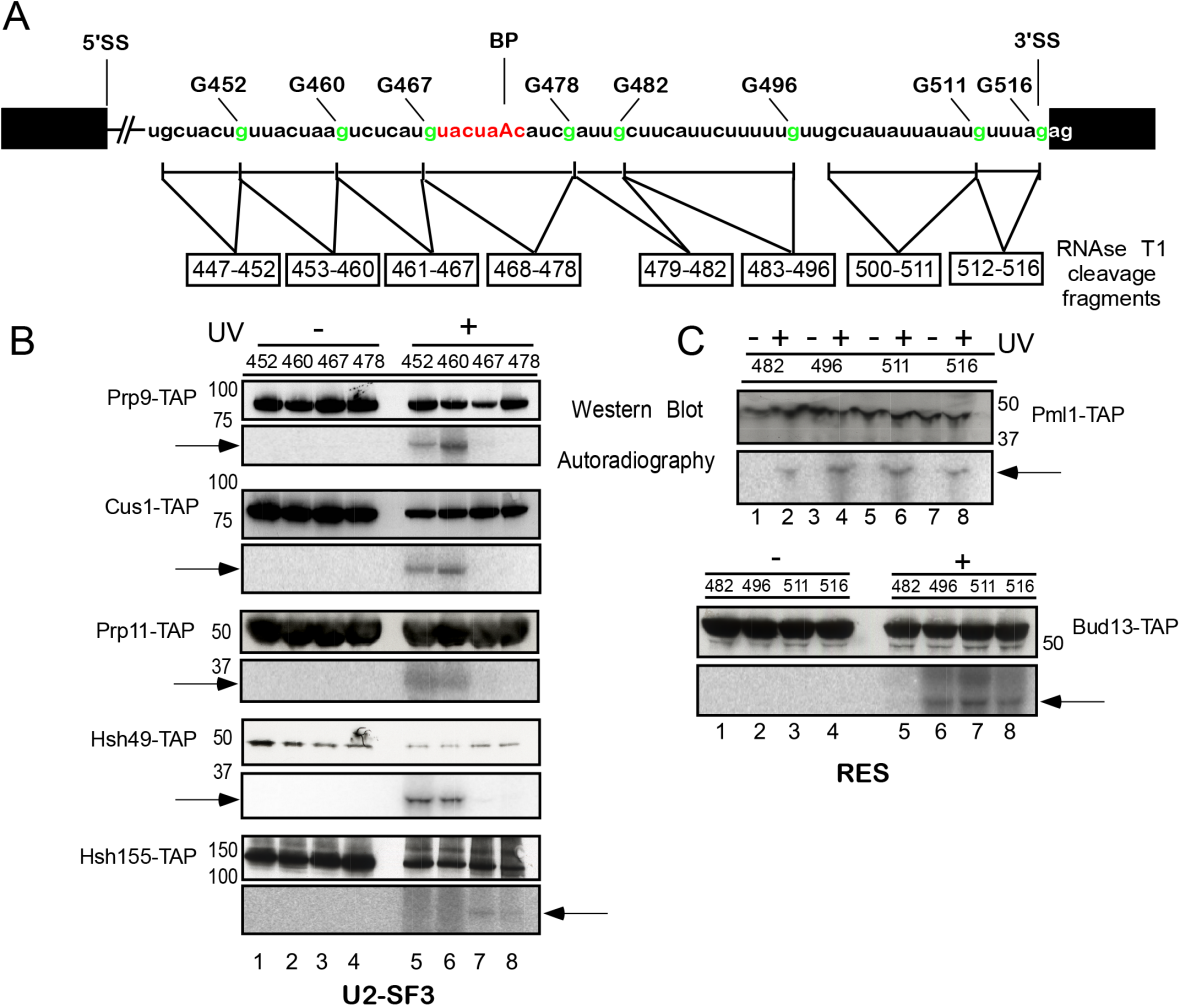
Site-specific UV crosslinking of U2 SF3a/b and RES complex proteins to the intron region around the branch-site in the yeast spliceosomal B^act ΔPrp2^ complex. (A). Schematic representation of site-specifically labeled pre-mRNAs carrying a single ^32^P-labeled phosphate 5’ of the guanosines shown in green. The RNA fragments remaining after digestion with RNase T1 are indicated by a box below the sequence. Spliceosomes were assembled on site-specifically labeled pre-mRNAs in splicing extracts of a yeast *prp2-1* strain carrying proteins tagged with the TAP-tag at their C termini. B^act ΔPrp2^ complexes were purified according to protocol 2 (S1 Text). (B). Purified B^act ΔPrp2^ complexes containing TAP-tagged U2 SF3a/b proteins were UV irradiated (+ lanes) or non-irradiated (–lanes). All samples were then digested with RNase T1 and subjected to immunoprecipitation with IgG Sepharose. Immunoprecipitates were analyzed by SDS-PAGE and subsequent western blotting with peroxidase anti-peroxidase (PAP) complex antibody (upper panel). The western blot shows bands of the expected size of the U2 proteins indicated (note that the TAP-tag increases the size of a protein by ca. 21 kDa). The autoradiography of the membrane is shown in the lower panel. (C) As in (B), except that purified B^actΔPrp2^ complexes containing TAP-tagged RES complex proteins were used. The arrows indicate ^32^P-labeled RNA fragments crosslinked to the respective proteins.

We prepared eight different actin–pre-mRNA constructs, each of which harbored a single ^32^P label directly 5’ of a distinct guanosine residue in the neighborhood of the BS (G452–G516, summarized in Fig 2A **)**. For this purpose, full-length non-^32^P-labeled pre-mRNAs were cleaved into two pieces at a specific position by using a distinct DNA enzyme; after 5’ ^32^P-labeling of the 3' piece, the two fragments were ligated by using the DNA splint-directed RNA-ligation method of Moore and Sharp. In this way, full-length pre-mRNAs were recreated, each containing a ^32^P-label at the desired position [22,25,26] (S1B Fig and Methods for details).

Extracts from the *prp2-1* strain harboring the TAP-tagged versions of proteins were then used for assembly of yeast B^act^ complexes on each of the site-specifically ^32^P-labeled pre-mRNAs. The purity of B^act^ complexes was determined by analyzing the composition of their associated snRNAs and pre-mRNA (i.e. for the presence of U2, U5L, U5S and U6 snRNA and the absence of splicing intermediates of the pre-mRNA; S2 Fig). Each purified B^act^ complex was irradiated with UV light at 254 nm and disrupted by incubating at 70°C in 3% SDS. After complete digestion with RNase T1 (which cleaves 3’ of guanosine residues), the RNA fragments shown in Fig 2A were obtained, each of which contained a single radioactive phosphate 5’ of the terminal guanosine residue. We then immunoprecipitated TAP-tagged crosslinked proteins with IgG Sepharose beads and analyzed the immunoprecipitates by western blotting using the PAP complex (peroxidase-anti-peroxidase) (Fig 2B and 2C, upper panels, western blot). Autoradiography of the membrane revealed the ^32^P-labeled RNA fragment crosslinked to each precipitated protein (lower panels). Thus, we were able to assign a well-defined pre-mRNA region crosslinked to a known protein and could map the entire intron area spanning from nts 447–516.

We first analyzed the U2 SF3a/b proteins and initially focused on the region upstream and across the BS (Fig 2B). For the RES complex proteins we focused on the region downstream of the BS (Fig 2C) because our earlier results showed that Snu17 is in direct contact with this region [20]. Western blotting confirmed that U2 proteins were immunoprecipitated either before irradiation (–UV) or after it (+UV); however, when UV irradiation was omitted, ^32^P-labeled fragments were not precipitated with the proteins (Fig 2B, lower panels, autoradiography, lanes 1–4). After UV irradiation, the U2 proteins Prp9, Cus1, Prp11 and Hsh49 were found crosslinked to the pre-mRNA fragments ^32^P-labeled at the G452 and G460 positions (i.e. fragments 447–452 and 453–460; Fig 2B, lower panels, lanes 5 and 6). None of the U2 proteins analyzed crosslinked to the downstream fragments 461–467 and 468–478, with the exception of Hsh155, which crosslinked to the RNA region 461–467 and with lower intensity to the BS region 468–478 (Fig 2B, lanes 7 and 8). The U2 proteins Rse1 and Prp21 and the two small proteins Rds3 and Ysf3 did not crosslink to pre-mRNA. This is consistent with earlier reports that the putative human homologue of Rse1, SAP130, could not be crosslinked to pre-mRNA [15] and Rds3 did not bind RNA *in vitro* [27]. Taken together, these results indicate that the U2 proteins Prp9, Cus1, Prp11, Hsh49 and Hsh155 are in direct contact with the pre-mRNA in the B^act^ complex, and their interaction is confined to a 14-nt-long region of the intron upstream of the BS (447–460), with the exception of Hsh155, which interacts in addition with the intron downstream of the BS [19] (see also below).

Fig 2C shows a similar pull-down experiment performed with purified, irradiated and RNAse-T1-digested B^act^ complexes carrying TAP-tagged RES Pml1 and Bud13 proteins. Western blotting showed that Pml1-TAP and Bud13-TAP were immunoprecipitated both before and after UV-irradiation. Pml1-TAP crosslinked the most strongly to the pre-mRNA fragments 483–496 and 500–511, while Bud13-TAP crosslinked to the fragment 500–511 (Fig 2C, lower panels). Thus, Pml1 interacts directly with the 14-nt-long region of the intron downstream of the BS (483–496), and both Pml1 and Bud13 interact further downstream than Snu17 [20].

### Dynamic contacts of U2 SF3 proteins with the pre-mRNA upstream of the branch-site upon catalytic activation and step 1 catalysis

**Fig 3:**
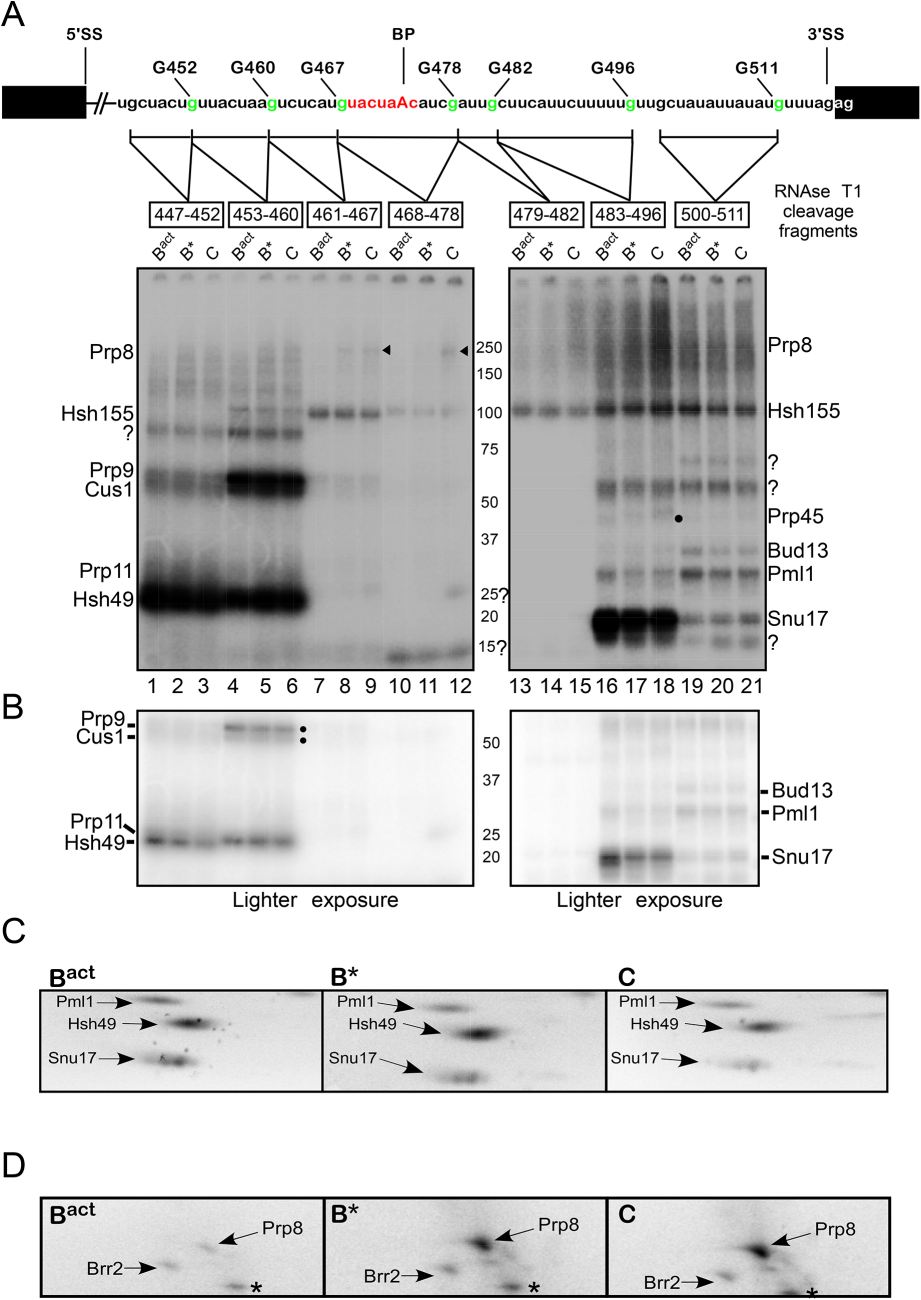
Dynamics of protein–pre-mRNA interactions around the branch-site. Schematic representation of site-specifically labeled pre-mRNAs as shown in Fig 2. B^actΔPrp2^ complexes were purified according to protocol 2 (S1 Text). Purified B^actΔPrp2^ and reconstituted B* and C complexes were UV irradiated, digested with RNase T1, and analyzed by SDS-PAGE electrophoresis. The amounts and molarity of the eluted spliceosomes were calculated on the basis of the specific activity of the pre-mRNA and equal molar quantity of B^act^, B* and C complexes were loaded onto the gel.The autoradiography of the gel is shown. Question marks indicate uncharacterized crosslinked proteins. The arrowheads point to crosslinked Prp8 and the dot indicates crosslinked Prp45. (B) Lighter exposure of the bottom half of the gel shown in panel (A). (C) Autoradiography of the bottom part of 2D gels comprising total proteins of purified, UV-irradiated and RNase-digested B^act^, B* and C complexes, respectively, which show the crosslinking intensities of Pml1, Hsh49 and Snu17 in each of the complexes. (D) As in C, only that the upper part of 2D gels is shown. The asterisk indicates a contaminant crosslinked protein.

Next, we expanded our analysis to the dynamics of protein–RNA interactions during catalytic activation and step 1 catalysis by using a purified yeast splicing system to investigate changes of UV crosslinking intensities in purified yeast spliceosomes stalled at specific assembly stages after B^act^, such as the B* and C complex stages [7]. B^act^ spliceosomes were prepared as above by incubating distinct site-specifically labeled actin pre-mRNAs (Fig 3A) with *prp2*-1 heat-inactivated splicing extracts in which proteins were untagged. Each B^act^ spliceosome was affinity-purified as above. One portion of B^act^ spliceosomes was complemented with ATP and recombinant Prp2 and Spp2 (whereby transformation of complex B^act^ to B* occurs) and one portion was complemented with ATP plus Prp2, Spp2 and Cwc25 (whereby transformation of complex B* to C occurs and step 1 is catalyzed). Spliceosomes were then further purified by glycerol-gradient sedimentation. The actual conversion from B^act^ to B* to C was analyzed for each purified complex by gel electrophoresis (S3 and S4 Figs). The presence of U2, U5 and U6 snRNA, and the total (or, for B*, nearly total) absence of splicing intermediates of the pre-mRNA confirmed the identity of the B^act^ and B* complexes, while the presence of step 1 products confirmed the identity of the C complex (S3C and S4C Figs). In addition, the efficiency of conversion of B^act^ to B* was determined by western-blot analysis, which revealed the nearly complete dissociation of the splicing factor Cwc24 from the B* complex during catalytic activation, as previously shown by MS and dual-color fluorescence cross-correlation spectroscopy (dcFCCS) [7,11]. The efficiency of conversion of B* to C complexes was monitored from the formation of step 1 splicing products, analyzed by 8% denaturing RNA PAGE and quantified by phosphorimager. The % of step 1 products (compared to the total RNA in a lane) was calculated to be ~40% (S3D and S4D Figs).

Peak fractions of purified B^act^, B* and C complexes were irradiated with 254-nm UV light and–after denaturation and digestion with RNase T1 –the crosslinked ^32^P-labeled proteins were analyzed by SDS-PAGE. The gel was subjected to autoradiography (Fig 3A). Each site-specifically labeled pre-mRNA showed a distinct crosslinking pattern, revealing bands of different intensities and masses. Fig 3A (lanes 1 and 4) shows different degrees of crosslinking of four proteins with sizes consistent with the apparent molecular masses of untagged Prp9, Cus1, Prp11 and Hsh49, which crosslinked to the pre-mRNA fragments 447–452 and 453–460 in the B^act^ complex. To ascertain that the four untagged crosslinked proteins corresponded to Prp9, Cus1, Prp11 and Hsh49 as shown in Fig 2B, we compared untagged and tagged proteins in parallel experiments. The molecular masses of tagged proteins are increased by a predicted 21kDa, along with the complete disappearance of the untagged version. S5 Fig lane 2 shows the patterns of untagged Hsh49, Prp11, Cus1 and Prp9 crosslinked to the pre-mRNA fragment ^32^P-labeled at G460 in the B^act^ complex. When the crosslinked proteins were compared with their tagged versions, we observed that Hsh49 shifted from 25kDa to ~50kDa (compare lanes 2 and 5, red arrow), Prp9 shifted from 60kDa to ~90kDa (compare lanes 2 and 3, yellow arrow), and Prp11 and Cus1 showed the expected size-shifts consistent with the addition of the TAP-tag (compare lane 2 with lanes 4 and 6; green and blue arrows, respectively). Similar comparisons were performed for the identification of Hsh155 and the RES proteins (S5B and S5C Fig). Taken together, these data allow assignment of the radioactive bands shown in Fig 3A to the proteins indicated (on the left and right of the gel).

The pattern in lane 1 of Fig 3A shows that Hsh49 crosslinked in highest yield to the ~6-nt-long region 447–452, whereas Prp9 and Cus1 crosslinked at low levels to the same fragment. Prp11 was not clearly distinguishable from Hsh49; however, as shown by a light exposure of the gel in Fig 3B, its crosslinking yield was very weak. Although the chemistry of the different sites in the RNA and proteins may affect the intensity of the crosslinks, these results do suggest that Prp9, Cus1 and Prp11 make no close contacts with this particular RNA region. Remarkably, however, the intensities of Prp9 and Cus1 crosslinks were much stronger in the ~8-nt-long region ^32^P-labeled further downstream (i.e. at G460) in the B^act^ complex, whereas crosslinks of Hsh49 (and Prp11) remained unchanged in this downstream ~8-nt-long region (Fig 3A, lane 4; see also Figs 3B and S5A for the crosslinking intensity of Prp11).

Intriguingly, upon conversion of the B^act^ to the B* and C complexes, crosslinks of Hsh49 to the ~6-nt-long region (447–452) were greatly reduced, as shown by light exposure of the gel in Fig 3B (lanes 1–3). Quantification of the intensity of Hsh49 crosslinks indicated that it was reduced by 40% and 80% in the B* and C complexes, respectively, relative to the B^act^ complex (S6A Fig). This indicates that remodeling of the spliceosome leads to a reduced interaction of Hsh49 with the ~6-nt-long region of the intron. Quantification of Prp11 crosslinks was difficult, as its weak signals did not resolve well from the strong signals of Hsh49. Interestingly, reduced crosslinking yield of Hsh49 to the downstream ~8-nt-long fragment (453–460) was also observed during spliceosome remodeling. However, the yield of crosslinks of Hsh49 to this region were reduced to a less significant extent in the B* and C complexes compared with the B^act^ complex (20% and 40%, respectively; S6A Fig). This indicates that Hsh49 maintains a relatively strong interaction with the ~8-nt-long region of the intron during catalytic activation and step 1 catalysis, compared to the upstream ~6-nt-long region. Similarly, during spliceosome remodeling the levels of crosslinking of Prp9 to the ~8-nt-long fragment (453–460) decreased by ~40% in both B* and C complexes compared with the B^act^ complex, indicating that also the binding site of Prp9 is destabilized (Figs 3A and 3B, lanes 4–6, and S6A). Although Cus1 was difficult to quantify, the pattern of its crosslinking seemed reduced to a similar extent (see Figs 3B, lanes 4–6, and S6A). Taken together, these results revealed that contacts of Hsh49, Prp11, Cus1 and Prp9 with adjacent regions of the intron were reduced to various degrees during the spliceosome’s conformational changes, indicating remodeling of the binding sites of these proteins.

To obtain independent evidence of the decrease over time of Hsh49 crosslinks, we performed 2D gel electrophoresis with UV-irradiated and RNase-digested B* and C complexes assembled on the 3’-region-labeled pre-mRNA, and compared the intensities of the signal from their crosslinked species with those observed in corresponding experiments with the B^act^ complex (Fig 3C). Consistently with the data shown in Fig 3A, the crosslinking level of Hsh49 to the 3’-region-labeled pre-mRNA was high in the B^act^ complex. However, the crosslinking level of Hsh49 decreased by ~10% in the B* complex and by more than 40% in the C complex relative to the B^act^ complex (Fig 3C), as determined by quantification of the radioactive spots (S6B Fig for quantification of Hsh49 in 2D gels). Thus, these results confirmed that contacts of Hsh49 with the 3’ regions of the intron were reduced during the spliceosome’s conformational changes, indicating remodeling of its binding sites.

A protein with the molecular mass of ~110 kDa, which was identified as Hsh155 (S5B and S5C Fig), crosslinked upstream and downstream of the BS. Hsh155 crosslinked strongest to the pre-mRNA fragment immediately upstream of the BS (461–467), and weakest to the BS fragment itself (468–478; Fig 3A, lanes 7–12), consistently with the result of the pull-down experiment shown in Fig 2B. Furthermore, we observed enhanced crosslinking of Hsh155 to the entire region downstream of the BS (Fig 3A, lanes 13–21). These results indicate that Hsh155 is in contact with a large 50-nt-long region of the intron and it spans the BS; however, its pattern of interaction with the intron does not seem to change significantly during remodeling of the B^act^ to B* and C complexes, with a decrease in the yield of crosslinking of only ~20% (S6A Fig).

There were also additional crosslinks that were not compatible with any obvious U2 snRNP protein equivalent (Fig 3A, marked with question marks). This suggests that there are additional proteins that also contribute to the protein–pre-mRNA interaction network in this region. Of interest is a 25 kDa protein that crosslinked with low intensity to the 11-nt-long BS fragment in the C complex (see below for the characterization of this protein). In addition, a ~15 kDa protein was observed that crosslinked to the BS fragment in B^act^, B* and C complexes; the identity of this protein could not be determined either by 2D gel electrophoresis or by tagging the small U2 proteins Rds3 or Ysf3. Furthermore, crosslinking of Prp8 and Prp45 was identified (see below for a detailed description).

### Dynamic contacts of all three RES subunits with the pre-mRNA downstream of the branch-site upon spliceosome remodeling

To shed some light on the dynamic interactions between the RES complex proteins and the intron, we investigated possible changes of their crosslinking pattern as described above for the U2 proteins. A protein of ~20 kDa was efficiently crosslinked to the pre-mRNA fragment 483–496 (Fig 3A, lanes 16–18). This protein, which was identified as Snu17 (see S5B and S5C Fig, lane 3), crosslinked strongest in the B^act^ complex; however, the intensity of crosslinking decreased by ~ 70% in the B* and C complexes relative to the B^act^ complex (Figs 3B and S6C), indicating that the interaction of Snu17 with the 14-nt-long region 483–496 is weakened after activation of the spliceosome by Prp2 (Fig 3A and 3B, lanes 16–18, see S7 Fig for an independent experiment). Analysis of Snu17 crosslinks by 2D gel electrophoresis, confirmed that the binding of Snu17 to the 3' region of the pre-mRNA is drastically reduced during spliceosome remodeling (Figs 3C and S6B for quantification of Snu17 in 2D gels)

The intensity of Pml1 crosslinking was much lower than that of Snu17 in the same region of the intron, suggesting that Pml1 either makes no close contacts with this region or simply binds in a manner unsuitable for forming crosslinks. Nonetheless, during transition of the spliceosome from B^act^ to B* the crosslinking intensity of Pml1 was reduced by ~50% and by another 10% from B* to C (Figs 3A, lanes 16–18 and S6C). A similar decrease in crosslinking intensity was confirmed by 2D gel electrophoresis (Figs 3C and S6B for quantification of Pml1 in 2D gels). Likewise, the levels of Pml1 and Bud13 crosslinks to the region further downstream (500–511) decreased by ~50–60% in the B* and C complexes relative to B^act^ (Figs 3A, lanes 19–21, and S6C). The intensity of Snu17 crosslinking was dramatically reduced in the 500–511 region of the intron, as compared with that upstream (483–496), indicating that the closest interaction of Snu17 with the intron is with the 14-nt-long region 483–496. This is consistent with results of our earlier pull-down experiments, which showed that Snu17 interacts directly with this region [20]. Taken together, these data indicate remodeling events involving RES protein contacts with the intron region downstream of the BS upon spliceosome conformational changes.

### Enhanced contacts of Prp8 with the branch-site and 3' splice site regions of the intron upon catalytic activation of the spliceosome by Prp2

A large protein with a molecular mass of ~250 kDa, which is the expected size for Prp8, crosslinked in very low yield to regions 461–467 and 468–478 of the intron (Fig 3A, lanes 7–12, indicated by arrowheads). Quantification of these crosslinked species revealed that Prp8 crosslinked to the region 461–467 of the intron: first in the B* complex and with increased level (by ~30%) in the post-step 1 spliceosome (Figs 3A, lanes 8 and 9, and S6C). Interestingly, Prp8 crosslinked weakly also to the BS sequence 468–478 in the C complex, indicating that during/after step 1 catalysis, Prp8 is favorably positioned for interaction with the BS (Fig 3A, lane 12). A stronger Prp8 crosslink was observed further downstream, to the 14-nt-long region 483–496 in the B* complex, the intensity of which was enhanced in the C complex (Fig 3A, lanes 16–18; see also S7 Fig for an independent experiment). Thus, our results indicate that Prp8 is favorably positioned for its interaction with the BS upon catalytic activation of the spliceosome by Prp2/Spp2, and with the 3’SS region upon subsequent step 1 catalysis by Cwc25.

Again independent evidence of the temporal increase of Prp8 crosslinks was obtained by 2D gel electrophoresis performed with UV-irradiated and RNase-digested B* and C complexes assembled on the 3’-region-labeled pre-mRNA, and the intensities of their crosslinked species was compared with those observed in the B^act^ complex (Fig 3D). Consistent with the data shown in Fig 3A, the crosslinking level of Prp8 to the 3’-region-labeled pre-mRNA was very low in the B^act^ complex, indicating that Prp8 makes no close contacts with the 3' region of the intron before catalytic activation by Prp2/Spp2. However, the crosslinking level of Prp8 increased more than 60% in the B* complex and even more than 90% in the C complex relative to the B^act^ complex (Figs 3D and S6B). Taken together, these results indicate that contacts of Prp8 with the BS and 3’SS regions begin during/after the catalytic activation by Prp2/Spp2 and the interaction with the 3'SS is enhanced after step 1 catalysis promoted by Cwc25, and are consistent with previous results that showed contacts of Prp8 with the 3'SS subsequent to step 1 catalysis in yeast extracts [28–31].

### Cwc25 is in contact with the branch-site region of the pre-mRNA in the C complex

**Fig 4:**
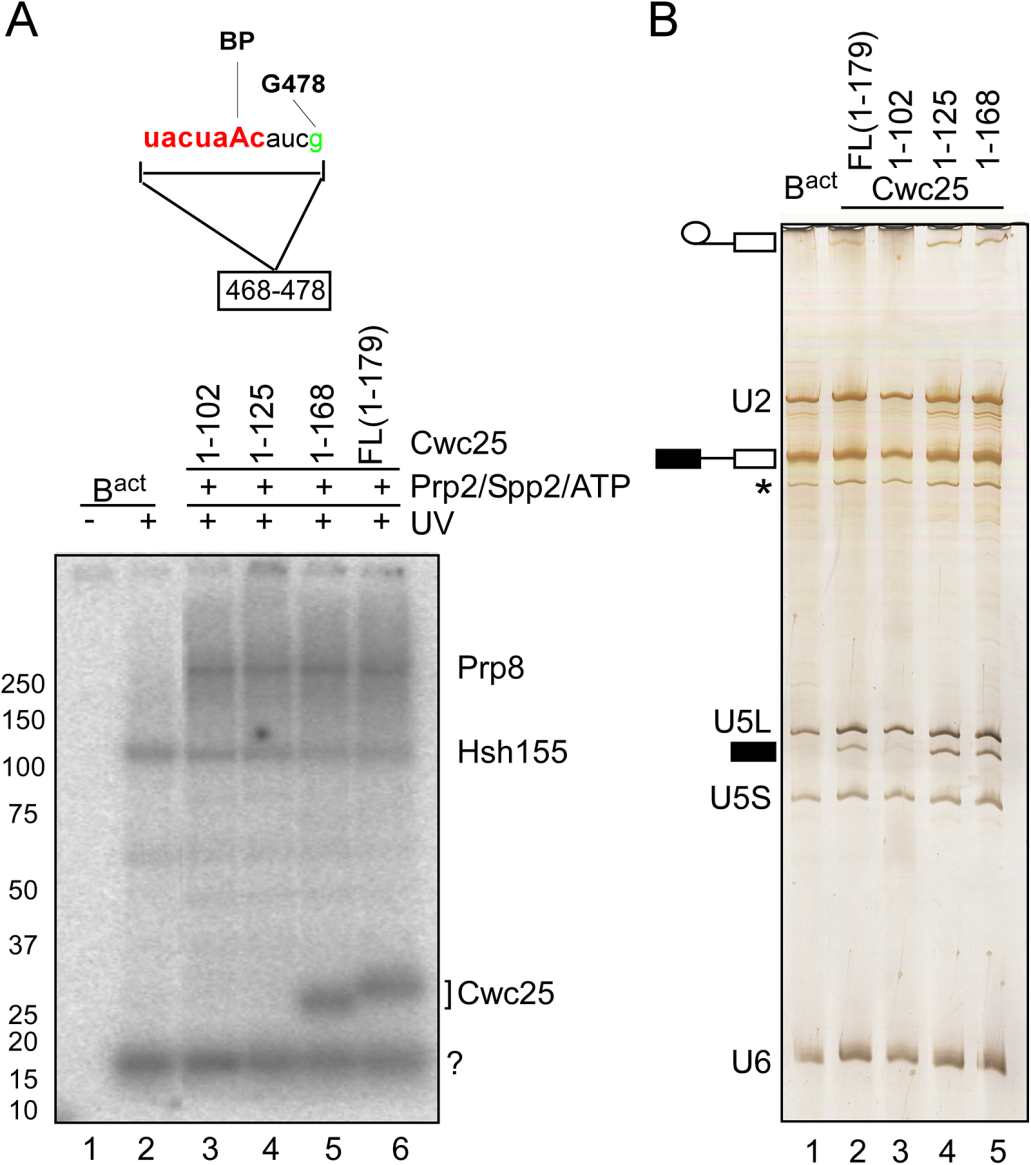
Cwc25 crosslinks within the branch-site region in the post-step 1 spliceosome. (A) Detail of site-specifically labeled pre-mRNAs carrying a single ^32^P-labeled phosphate 5’ of the guanosine in position 478 of the intron. The RNA fragment remaining after digestion with RNase T1 is indicated. Spliceosomes were assembled on the site-specifically labeled pre-mRNA, in extracts of a yeast *prp2-1* strain. B^actΔPrp2^ complexes were purified according to protocol 2 (S1 Text). Purified B^actΔPrp2^ complexes were not irradiated (lane 1) or UV-irradiated (lane 2) or complemented with recombinant Prp2, Spp2 and full-length Cwc25 or truncated variants thereof, in the presence of ATP, to generate C complexes. All samples were UV-irradiated and then digested with RNase T1, and analyzed by SDS-PAGE electrophoresis. The autoradiography of the gel is shown. The question mark indicates an uncharacterized crosslinked protein. (B) The RNA isolated from the complexes shown in (A) before UV irradiation, was analyzed by denaturing gel and stained with silver. The presence of U2, U5L, U5S and U6 snRNA, and the absence of splicing intermediates of the pre-mRNA confirmed B^act^ complexes identity (lane 1). The presence of step 1 products established the identity of the C complex. The asterisk indicates the presence of a small amount of U1 snRNA. Symbols for pre-mRNA, splicing intermediates and products are indicated on the left: the 5' exon in black, intron as a thin black line, 3' exon in white.

To determine the identity of the 25kDa protein, which crosslinked to the BS fragment 468–478 in the post-step 1 spliceosome, we used recombinant full-length Cwc25 and truncated variants thereof in reconstitution of the C complex. Reconstituted C complexes were UV-irradiated and RNAse-T1-digested as above. Fig 4A shows that recombinant Cwc25 crosslinked to the BS fragment in the C complex (lane 6). A truncated variant of Cwc25 (residues 1–168), lacking 11 amino acids at the C-terminus, showed a similar crosslinking yield (lane 5), In contrast, the two variants Cwc25 1–102 and 1–125, lacking 77 and 54 amino acids at their C-termini, did not crosslink to this region (lanes 3 and 4). Consistently with previous observations [32], this experiment demonstrates that Cwc25 is in contact with the BS sequence and that at least the N-terminal 168 amino acids of Cwc25 are needed for this.

Intriguingly, the addition of the truncated version of Cwc25 1–168 (Fig 4A, lane 5) to B* spliceosomes promoted step 1 catalysis, which was even more efficient than that observed with the full-length version (Fig 4B, compare lanes 5 and 2). Surprisingly, Cwc25 1–125 promoted step 1 catalysis even in the absence of RNA crosslinking (Fig 4B and 4A lanes 4), indicating that Cwc25’s activity in promoting step 1 can be uncoupled from its activity in RNA-binding/crosslinking. This result suggests that Cwc25 1–125 may still interact with one or more proteins in the neighborhood of the BS and thus render the microenvironment of the catalytic center favorable for step 1 catalysis. Candidate proteins for interaction with Cwc25 are Prp8 and Hsh155, which are shown here to crosslink to the BS region concomitantly with Cwc25 (Fig 4A). Furthermore, Yju2 may also interact with Cwc25, as it was previously shown to be involved in recruiting Cwc25 to the spliceosome [9].

### The NTC-related proteins Prp45 and Prp46 contact the region near the 3’ splice site

**Fig 5:**
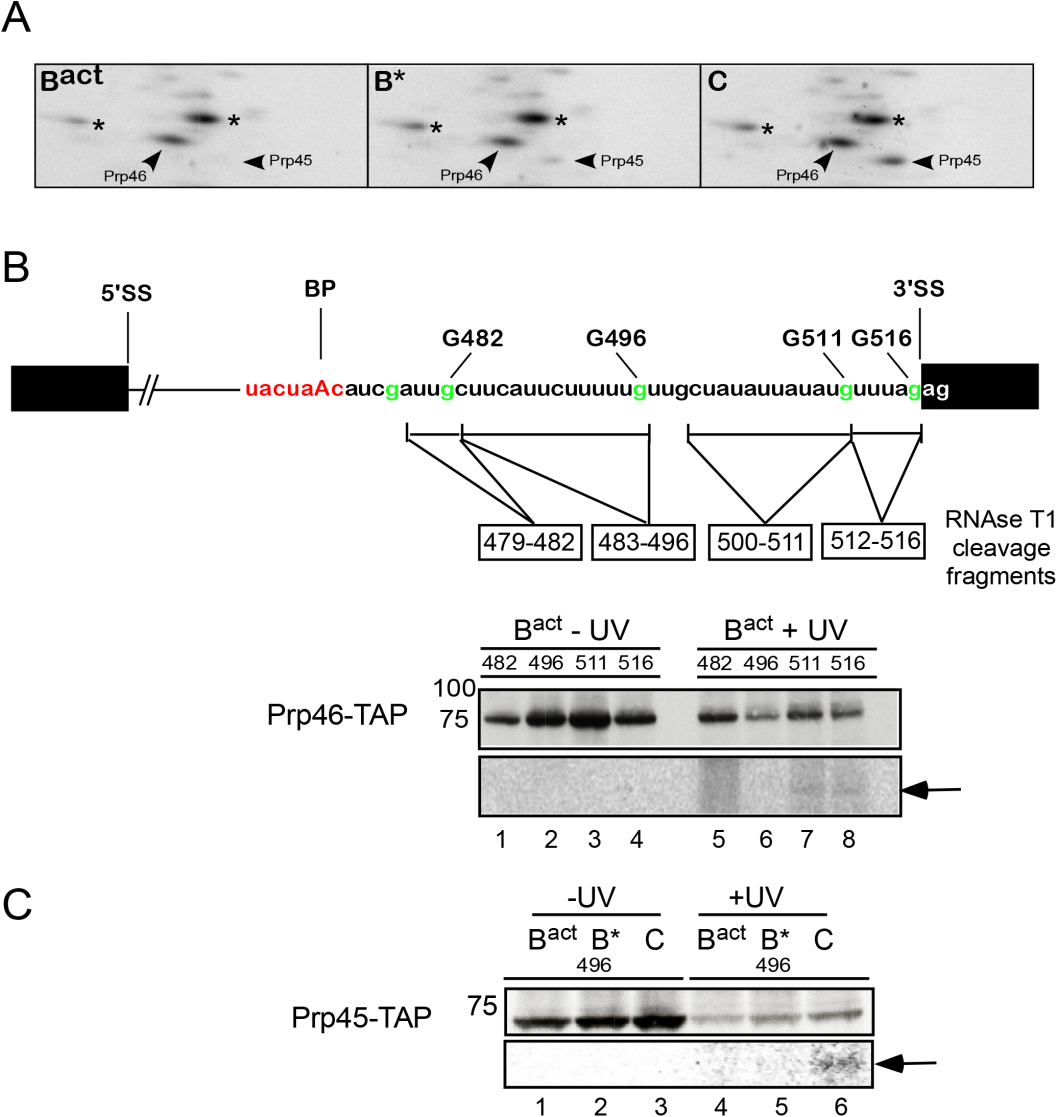
Interaction of Prp46 and Prp45 with the region around the 3’SS during catalytic activation and step 1 catalysis. (A) Autoradiography of portions of 2D gels comprising total proteins of purified, UV-irradiated and RNase digested B^act^, B* and C complexes, respectively. The asterisks indicate contaminant crosslinked proteins. (B) Schematic representation of site-specifically labeled pre-mRNAs as shown in Fig 2. B^actΔPrp2^ spliceosomes were assembled on pre-mRNAs site-specifically labeled 5’ of the different G nucleotides as shown, in splicing extracts of a yeast *prp2-1* strain carrying Prp46 tagged with the TAP-tag and were purified according to protocol 2 (S1 Text). Spliceosomes were then UV irradiated, digested with RNase T1, and subjected to immunoprecipitation with IgG Sepharose. Immunoprecipitates were analyzed by SDS-PAGE and subsequent western blotting as above (upper panel). The western blot shows a band of the expected size of Prp46-TAP. The autoradiography of the membrane is shown in the lower panel and the RNA fragments crosslinked to Prp46-TAP are marked by an arrow. (C) as in (B) B^act^ spliceosomes were assembled on the pre-mRNA site-specifically labeled 5’ of the G nucleotide at position 496, in splicing extracts of a yeast *prp2-1* strain carrying Prp45 tagged with the TAP-tag. Upon addition of Prp2/Spp2 and Cwc25, B* and C complexes were obtained. The western blot shows a band of the expected size of Prp45-TAP. The RNA fragment crosslinked to Prp45-TAP is marked by an arrow.

In addition to the NTC, two splicing factors, namely Prp45 and Prp46 [33] that interact with components of the NTC, and whose function is related to NTC in human and yeast, were also observed in the 2D gel carried out with B^act^ spliceosomes (Fig 1B). We observed that Prp45 did not crosslink in B^act^ complexes assembled on the 3’-region-labeled pre-mRNA after irradiation with UV light (Fig 1C), in contrast, Prp46, which was previously shown to interact with Prp45 *in vitro* and *in vivo* [33], crosslinked in high yield to the 3' end of the intron in B^act^ spliceosomes (Fig 1C). To determine whether this crosslink was retained during spliceosome remodeling, we prepared 2D gels from crosslinked, RNase-digested B* and C complexes assembled on the 3’-region-labeled pre-mRNA. Fig 5A shows that the crosslink of Prp46 was preserved with a similar yield in the B* complex but it increased by ~20% in the C complex (S6B Fig). This indicates that Prp46 remains in contact with the 3’ end of the intron during remodeling of the B^act^ to B* and to C complexes. To map more precisely the RNA interaction site of Prp46, we performed UV crosslinking of B^act^ spliceosomes assembled on site-specifically labeled pre-mRNAs in Prp46-TAP extract (Fig 5B). After pull-down, we observed that Prp46-TAP crosslinked weakly to both RNA fragments labeled at G511 and G516 (Fig 5B, lanes 7 and 8). Despite the strong crosslink of Prp46 observed in 2D gels obtained from the B^act^ complex (Fig 5A), we detected low levels of Prp46 crosslinks in the B^act^ complex assembled on each of the two site-specifically labeled pre-mRNAs (lanes 7 and 8). Taken together, these results suggest that the prominent crosslink of Prp46 in the 2D gel may be due either (i) to an additional crosslinked protein co-migrating with Prp46 or (ii) to interaction with a region located further upstream than the region 479–482. Alternatively, or additionally, the TAP-tag fused to Prp46 may prevent efficient crosslinking of Prp46 to the intron (Fig 5B).

Although Prp45 did not crosslink in B^act^ complexes assembled on the 3’-region-labeled pre-mRNA after UV irradiation (Figs 1C and 5A), we nonetheless observed a protein with the expected size of Prp45 (i.e. ~42kDa), which crosslinked in low yield to the fragment 483–496, in C complexes (Figs 3A, lane 18 marked by a dot, and S7). The identity of Prp45 was determined by pull-down of crosslinked and T1-digested complexes containing Prp45-TAP, assembled on pre-mRNA site-specifically labeled at G496 (Fig 5C). Prp45-TAP crosslinked (with low intensity) only in the C complex (lane 6), indicating that Prp45 makes contact with the region of the intron 483–496 after step 1 catalysis. Independent evidence that Prp45 contacts the pre-mRNA upon step 1 catalysis was obtained again from the analysis of 2D gels obtained from crosslinked B* and C complexes that were assembled on the 3’-region-labeled pre-mRNA (Fig 5A). Prp45 crosslinked in the B* complex at low levels, yet the intensity of this crosslinked species increased more than 80% in the C complex (Figs 5A, S6B and S7). This result suggests a temporal interaction of Prp45 with the intron's region near the 3' SS upstream of Prp46, and indicates that Prp45 contacts the 3’ end of the intron after/during step 1 catalysis.

## Discussion

Here we have investigated pre-mRNA–protein contact sites in affinity-purified yeast B^act^ spliceosomes by UV crosslinking. A number of crosslinked proteins of the U2 snRNP, including the SF3a subunits Prp9 and Prp11 and the SF3b proteins Cus1, Hsh49 and Hsh155, as well as RES complex proteins and their contact sites on the pre-mRNA intron, could be precisely assigned by performing crosslinking followed by 2D gel electrophoresis and immunoprecipitation. Taken together, the results indicate that the branch-site region is contacted at several positions, apparently over its entire length, by proteins. A similar investigation was carried out with affinity-purified spliceosomal B* and C complexes. The results presented here provide much-needed information regarding the spliceosomal pre-mRNA–protein network, and they show for the first time that also yeast U2 SF3a/b proteins, as their human counterpart, are tightly anchored around the BS region. They also provide insight into the dynamics of pre-mRNA–protein interactions involving Cwc25, Prp8 and Prp45 within the spliceosome upon its conversion into the B* (i.e., catalytically activated) complex and during the subsequent conversion of the latter into the C complex (i.e., the step 1 spliceosome).

### U2 SF3a and SF3b interactions with the intron region surrounding the BS are conserved in evolution

In the human system, the U2 protein-pre-mRNA interactions are already established in the early A complex but remain in the rearranged, activated spliceosome [15,34]. Here, we analyzed U2 protein–pre-mRNA interactions initially in purified B^act^ complexes, likely our data obtained with B^act^ complexes apply also to earlier complexes (i.e. A and B complexes), which for practical reasons were not analyzed here.

Using a combination of UV crosslinking and immunoprecipitation of TAP-tagged proteins, we were able to assign a number of U2 snRNP proteins crosslinked to specific sites using pre-mRNAs that were labeled at specific positions by a combination of DNA enzymes cleavage and splint-directed ligation [22]. Site-specific labeling of the RNA with ^32^P is a much more promising approach for UV crosslinking studies, because the RNA–protein interaction site can be precisely mapped on the RNA. For the first time, we were able to use purified yeast spliceosomes to perform a comprehensive protein–pre-mRNA interaction analysis and thus to assign a well-defined RNA region crosslinked to a known protein. In this way we were able to map an extensive area, spanning a 70-nt-long region of the intron.

Consistent with previous studies with human spliceosomal complexes, crosslinking sites involving the yeast U2 SF3a proteins Prp9 and Prp11, as well as SF3b proteins Cus1 and Hsh49, were observed within a 14-nt-long region upstream of the BS of affinity-purified B^act^ complexes (Fig 2B). Furthermore, consistent with previous results obtained in yeast [19] and human [14] spliceosomes, immunoprecipitation revealed contacts between a region located further downstream (surrounding the BS) and SF3b Hsh155. These results provide evidence that the region directly upstream of the BS, and surrounding it, is the main interaction platform of the yeast U2 snRNP proteins. Likewise all human U2 snRNP-associated SAPs, except for SAP130, were found in direct contact with a 20-nt-long region upstream of the BS in the isolated spliceosomal complexes A, B, and C [34,35] and SF3b155 was also found to bind to a site downstream of the BS [14]. Thus, our data furthermore suggest that U2 protein-pre-mRNA interactions with the regions upstream and downstream of the BS are conserved between yeast and human (S1 Table). Furthermore, consistent with earlier findings [15], an oligoribonucleotide complementary to the 14-nt-long region upstream of the BS inhibits formation of the yeast A, B and B^act^ complexes (S8 Fig). Thus, interactions of SF3a and SF3b with the pre-mRNA appear to be a prerequisite for pre-spliceosome formation also in yeast, indicating that the perfect complementarity between the BS sequence and the U2 snRNA is not sufficient to anchor the U2 snRNP to the BS sequence, and that stable binding of U2 is largely dependent on U2 protein–pre-mRNA interactions.

### The RES proteins Pml1 and Bud13 make weak contacts with the intron between the branch-site and the 3’ splice site

The RES complex is a conserved, spliceosome-associated module that has been shown to enhance splicing of a subset of transcripts and to promote the nuclear retention of unspliced pre-mRNAs in yeast [10]. Furthermore, it was shown to be required for efficient splicing of TAN1 pre-mRNA, and the intron sequence between the 5'SS and the BS was necessary and sufficient to mediate dependence upon RES [36]. Here, we identified low-yield crosslinks between the BS and the 3’SS of both Pml1 and Bud13. Consistent with results from our earlier studies [20], we show that the other RES complex protein, Snu17, is in direct contact with the pre-mRNA in the region between the BS and the 3’SS within a 14-nt-long RNA stretch upstream of the G nucleotide at position 496. Our data do not conflict with the result observed with the TAN1 pre-mRNA because the requirement of TAN1 intron nts upstream of the BS for RES dependence could be transient, and an interaction may occur earlier during spliceosome assembly.

**Fig 6:**
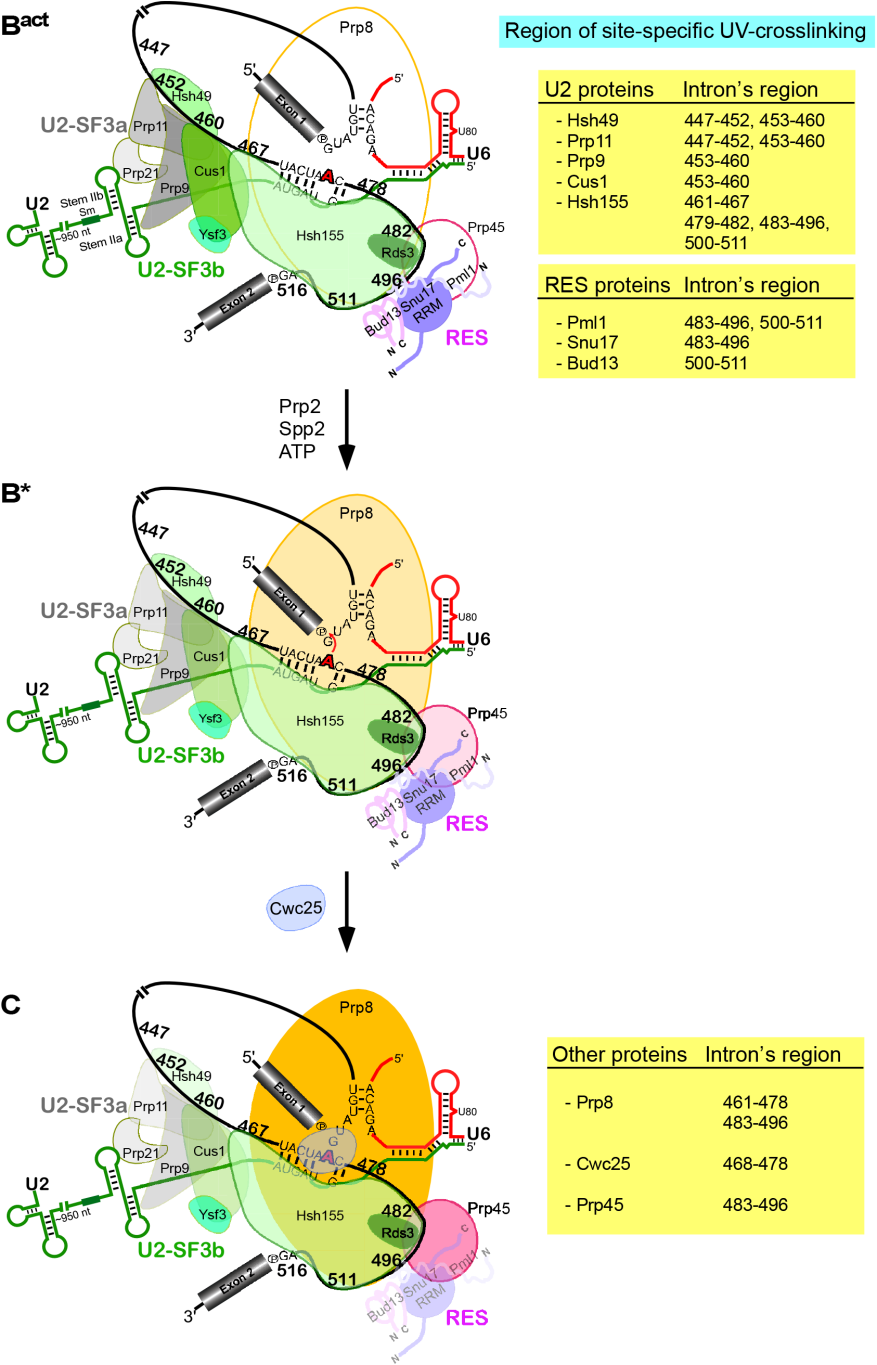
Summary of site-specific UV crosslinking of proteins to the intron region around the branch-site in the B^act^ complex and their dynamics following the conversion of B^act^ into the B* complex (i.e., catalytically activated) and during the subsequent conversion of the latter into the C complex (i.e., the step 1 spliceosome). Schematic representation of the secondary-structure model of U2/U6/pre-mRNA interactions in the B^act^ complex. The branch point A is indicated by a red bold letter. Sites in the pre-mRNA's intron crosslinked to the U2-SF3a (grey), U2-SF3b (green), RES complex proteins [(shades of purple, represented schematically according to [20,38,40]], Prp8, Cwc25 and Prp45 are indicated by the number of the site-specifically labeled guanosines. The regions of site-specific UV-crosslinking are also summarized on the Tables on the right. Changes in crosslinking yields upon conversion of B^act^ to B* to C are highlighted by changes in color intensities.

Our observation of direct contact between Snu17 and the intron is in agreement with earlier reports showing that Snu17 consists primarily of a RRM, which is probably involved in contacting the RNA. It was also reported that the RRM of Snu17 is atypical and acts as a central binding platform that provides two separate interaction surfaces, which interact with disordered parts of Bud13 and Pml1 at the same time [37–39]. While Bud13 and Pml1 do not harbor typical RNA-binding domains, Bud13 contains a conserved lysine-rich region that might bind RNA. In addition, Pml1p or U2 proteins interacting with RES in the spliceosome (such as the SF3b Hsh155) might facilitate the recognition of RNA by RES. A recent NMR solution structure of the core of the RES complex revealed that complex formation leads to intricate folding of the three components that stabilize the RRM fold of Snu17 upon binding of Bud13 and Pml1, while RNA binding efficiency is increased [20]. Taken together, our results indicate that Snu17 crosslinks directly to the intron between the BS and the 3’SS in the B^act^ complex, while Pml1 and Bud13 may make contact with the intron through their elaborated interconnection with Snu17, but they may be in a conformation that does not favor the formation of UV-induced crosslinks (see Fig 6 for a summary and S2 Table).

As Hsh155 is in contact with nucleotides of the pre-mRNA between the BS and 3’SS (Fig 3), which are also in contact with all three components of RES, this indicates that Hsh155 and the RES proteins are in close proximity to one another in the B^act^ spliceosome. This is consistent with earlier studies showing by a yeast two-hybrid screen and co-immunoprecipitation experiments that Snu17 interacts with U2 SF3b proteins [18]. Furthermore, the RES complex subunit Snu17 was shown to bind to the U2 snRNP [41]. Taken together, all these studies indicate that there is a direct interconnection between RES, the U2 SF3b proteins and the pre-mRNA downstream of the BS.

### Evidence for subtle changes of the U2 and RES protein contacts with the pre-mRNA upon spliceosome remodeling by Prp2 and subsequent step 1 catalysis

Examination of U2 protein–pre-mRNA interactions in purified spliceosomal complexes stalled after catalytic activation by Prp2/Spp2 (B* complex) and subsequent step 1 catalysis by Cwc25 (to form the C complex), revealed that the spliceosome structure involving the region of the intron upstream of the BS and the SF3 proteins Prp9, Hsh49 and Cus1 undergoes a conformational change during spliceosome activation and subsequent step 1 catalysis. That is, crosslinks of Prp9, Hsh49 and Cus1 were significantly reduced in both spliceosomal complexes compared with those observed with the B^act^ complex, indicating that binding to pre-mRNA of these proteins is destabilized after ATP hydrolysis by Prp2. This is consistent with the remodeling of the structure of the catalytic core of the spliceosome near the BS upon nucleophile attack on the 5' SS phosphodiester bond during step 1 catalysis. That is, alterations in U2 protein binding are probably due to conformational changes that destabilize the interactions of these proteins with the pre-mRNA upstream of the BS concomitant with step 1. Intriguingly, our data reveal that contacts of Hsh49, Cus1 and Prp9 (and to a lesser extent Prp11) with two adjacent short regions of the intron upstream of the BS were reduced, to different degrees, during the remodeling of the spliceosome. This indicates that SF3 proteins remain in contact with the intron upstream of the BS even after step 1 catalysis, yet their binding affinity to the pre-mRNA is significantly reduced at a certain site and partially abolished at another. Our results further indicate that the complete set of U2 proteins remains in contact with the U2 snRNA via protein–RNA or protein–protein interaction. Indeed, it was shown that the U2 snRNP is released from the intron-lariat spliceosome *in vitro* as an integral snRNP, indicating that it remains intact during the entire splicing cycle and that none of its proteins are lost under physiological conditions *in vitro* [24].

Furthermore, as suggested by their decreased efficiency of crosslinking during spliceosome remodeling (Fig 3), the binding of the RES complex as a whole is reduced. Remodeling events involving the RES complex proteins are intriguing because the same stretch of the intron is also bound by Prp2 and is essential for Prp2- and Spp2-mediated catalytic activation [8,42]. Indeed, Prp2 was crosslinked to the same nucleotides of the intron as the RES proteins [8]; thus, it may be possible that Prp2 recognizes this stretch of RNA "productively" only when it is in contact with the RES proteins. The RES complex could be recognized as an entry point or primary target by Prp2/Spp2 to initiate translocations along the intron [42], thereby destabilizing RNA-bound proteins and acting as a classical RNPase. Alternatively, it was recently suggested that Prp2, in addition to binding the intron, is probably involved in several protein–protein interactions in the spliceosome [8]. This would lead to the formation of a relay system that could transmit a power stroke within the motor module of Prp2/ATP, through the various anchor points that Prp2 shares with other components of the spliceosome [8]. Thus, the RES–the binding of which is destabilized upon Prp2/Spp2-mediated B* formation [11] (and this work)–could be an important primary element of this communication system. Importantly, it was recently reported that a prp2 mutant was suppressed by deletion of *PML1*, indicating that Pml1 stabilizes an interaction that Prp2 destabilizes [43].

### Prp8 is favorably positioned for its interaction with the branch-site and the 3’ splice-site regions of the intron after catalytic activation of the spliceosome by Prp2

To date, Prp8 is the only spliceosomal protein that has been shown to crosslink to all the three regions in pre-mRNA that are required for splicing (5’SS, 3’SS, and BS), as well as to U5 and U6 snRNAs [44,45]. Here, by 2D gel electrophoresis of affinity-purified B^act^ complexes, we provide evidence that Prp8 –although already stably associated with B^act^–makes no close contacts with the 3’ region of the intron before catalytic activation by Prp2/Spp2. Using affinity-purified spliceosomes stalled at the B* and C stages, we show that conformational changes leading to step 1 catalysis bring Prp8 to a position near the BS and the 3’SS (Figs 3 and S7) [28–31]. That is, remodeling at the catalytic core of the spliceosome accompanies stabilization of Prp8–pre-mRNA contacts. Intriguingly, previous work showed that high-affinity binding sites are created in the B* complex–also for additional factors required for step 1 catalysis such as Yju2 and Cwc25 –during catalytic activation [11]. Thus, the ATP-dependent Prp2-driven activation of the spliceosome leads not only to reduced contacts with the pre-mRNA of U2 and RES proteins, but also to stabilization of other protein–pre-mRNA interactions by promoting direct contact with the pre-mRNA. Taken together, these results provide new insight into the dynamics of protein–pre-mRNA interactions (simultaneous reduction of some and enhancement of others) within the spliceosome during its catalytic activation and catalysis.

### The step-1-promoting and RNA-binding activities of Cwc25 are independent

Step 1 catalysis cannot occur efficiently without Cwc25 [7,9]. After Prp2-mediated catalytic activation of the spliceosome, a strong binding site is created on the B* spliceosome for the step 1 factor Cwc25. While Cwc25 only shows background binding to complex B^act^, its binding to complex B* has a *K*_d_ value in the subnanomolar range [11]. Consistent with the enhanced binding of Cwc25 upon the action of Prp2, we show here that Cwc25 crosslinks to the 11-nt-long BS fragment. These data are in agreement with earlier reports showing that Cwc25 crosslinks to the intron sequence three bases downstream of the BS [32]. Interestingly, using truncated versions of recombinant Cwc25 for reconstitution of C complexes, we observed that Cwc25 1–125 (lacking 54 amino acids at its C-terminus) promoted step 1 catalysis even in the absence of RNA crosslinking, indicating that Cwc25’s step-1-promoting activity is not coupled to its pre-mRNA interacting activity. This indicates that contacts of Cwc25 1–125 would theoretically occur with one or more proteins in the proximity of the BS, thus making the microenvironment of the catalytic center suitable for step 1 catalysis. This would be consistent with Cwc25 being one of the intrinsically disordered proteins, which are highly connected or “promiscuous” proteins that undergo several simultaneous or sequential interactions and use regions of disorder as a scaffold for assembling an interacting group of proteins [46]. Thus, Cwc25 might act as an important hub in the catalytic center of the spliceosome. Indeed, we observed Cwc25’s contacts in the BS region of C complexes concomitant with enhanced crosslinking of Prp8 and Prp45 to the same or a slightly downstream region (Figs 3–5), suggesting that Cwc25 co-ordinates the catalytic center through protein–protein interaction.

### Prp45 is favorably positioned for its interaction with the 3’ splice site regions of the intron after step 1 catalysis

We showed that Prp45 contacts the pre-mRNA only after step 1 catalysis (Figs 5C and S7), although it is already associated with the spliceosome at the B^act^ stage (Fig 1B). Earlier results showed that, in addition to their interaction in two-hybrid screens, Prp45 and Prp46 interact *in vitro*, most probably through direct protein–protein contact [33]. Here, Prp45 crosslinked to the pre-mRNA region of the intron 483–496 and Prp46 to the region immediately downstream (i.e. 500–516), indicating that the two proteins are also in close contact during spliceosome remodeling. Interestingly, Prp45 crosslinked during or after step 1 catalysis to the same pre-mRNA region of the intron (i.e. 483–496) where Prp8 was also found to crosslink with highest yield (S7 Fig), indicating that profound remodeling events involving this region occur. Indeed, a simultaneous reduction of Snu17 and Pml1 contacts was also observed (Figs 6 and S7 for a summary).

The contact of Prp45 to this region is consistent with earlier work that showed that Prp45 interacts with Prp22, a DEAH-box RNA helicase involved in spliceosome disassembly [33,47], which was also crosslinked to the 3’SS in post-step 1 spliceosomes [48]. In addition, a temperature-sensitive allele of Prp45 was shown to be synthetically lethal with alleles of several second-step splicing factors (Slu7, Prp17, Prp18 and Prp22) and with several NTC components. Thus, Prp45 may be required for Prp22 as well as the recruitment or stabilization of additional step 2 factors, and the positioning of Prp45 close to the 3’SS after step 1 may determine the timing of this event. The timing of the direct interaction of Prp45 with the pre-mRNA may explain the contribution of this protein to step 2 catalysis: this could be effected either (i) by participating in maintaining the step 2 conformation, or (ii) by binding and regulating the Prp22 ATPase/translocase activity [47].

## Materials and Methods

### Site-specific cleavage of RNA with DNA enzymes

In a first step, actin pre-mRNA was prepared by transcription *in vitro* with T7 RNA polymerase (S1 Text). The transcription reaction was not gel-purified, but instead was precipitated with ethanol. After washing twice with 70% ethanol, the precipitated RNA was dried, dissolved in 50μl CE buffer (10 mM cacodylic acid-KOH, pH 7.0, 0.2 mM EDTA-KOH, pH 8) or water and applied to a G 50 spin column (GE Healthcare). The eluted RNA was then subjected to DNA enzyme cleavage, essentially as described previously [25]. First, a threefold molar excess of the DNA enzyme over the pre-mRNA was added to the reaction mixture. The solution was then adjusted to 15 mM NaCl and 5 mM TRIS-HCl, pH 7.7. After denaturation at 70°C for 2 min the mixture was kept at room temperature for 5 min. Finally, 150 mM NaCl, 50 mM Tris-HCl, pH 7.7 and 2 mM of both MgCl_2_ and MnCl_2_ were added and the mixture was incubated at 30°C for 3 hrs. To remove the cyclic phosphate produced at the 3’ end of the 5’ fragment by the DNA enzyme, the intrinsic 3'-phosphatase activity at low ATP concentration of T4 polynucleotide kinase (T4 PNK) was used [26]. The reaction was supplemented with 2 unites/μl T4 PNK, PNK buffer and 0.4 mM ATP and incubated for 1 h at 37°C [26]. The RNA digestion fragments were gel-purified as described for *in vitro* transcriptions (S1 Text).

### 5’ ^32^P labeling with T4 PNK

For the production of site-specifically labeled pre-mRNAs, the purified 3’ pre-mRNA fragment created by DNA enzyme cleavage was 5’-phosphorylated with 2 μM γ-^32^P ATP, T4-PNK buffer, 2 units/μl RNAsin and 1 unit/μl T4-PNK in a total volume of 20 μl or more, depending on the experiment. The reaction mixture was incubated for 1 h at 37°C and then purified by using a G 50 spin column, followed by phenol-chloroform-isoamyl alcohol (PCI) extraction and ethanol precipitation.

### DNA splint-directed Moore and Sharp RNA ligation

RNA fragments were ligated by DNA splint-directed RNA ligation [22]. The 5’-ligation fragments were prepared by DNA enzyme cleavage followed by 3’-dephosphorylation as described above. For site-specific labeling, the 3’ ligation fragment was labeled at the 5’ end with γ-^32^P ATP as described above. For region-specific labeling, the 3’ fragment was produced by radioactive *in vitro* transcription using GMP as a starting nucleotide (S1 Text). The 5’ ligation fragment, the DNA splint and the 3’ ligation fragment were mixed in a 1.4:1.2:1 ratio. After addition of T4 DNA ligase buffer and water, the reaction was denatured for 2 min at 70°C and the sample was then cooled to 30°C at 6°C per min. Thereafter 1 mM ATP, 2 units/μl RNAsin and 3 units/μl T4 DNA ligase were added and the reaction was incubated for 3 hrs at 30°C. Finally, the ligation product was gel-purified. The efficiency of ligation was ~30–60%.

## Supporting Information

S1 Fig Synthesis of region- and site-specifically labeled pre-mRNA. (A) Experimental strategy used for the production of region-specifically labeled pre-mRNA. Upper panels. Left: Reaction mechanism of RNA cleavage by DNA enzymes (adapted from Silverman and Baum [25]). Right: Representation of the 8–17 deoxyribozyme. Base pairing between the target RNA and the recognition arms of the DNA enzymes and the supposed structure and sequence of the catalytic DNA loop are represented schematically (adapted from Silverman and Baum [25]). The pre-mRNA is represented schematically by lines indicating the intron and by rectangles indicating the exons. Radioactively labeled stretches are shown in green. (B) Experimental strategy used for the production of site-specifically labeled pre-mRNA. The pre-mRNA is represented schematically by lines indicating the intron and by rectangles indicating the exons. The ^32^P introduced by this procedure is shown in green.
(TIF)

S2 Fig Characterization of B^act^ complexes carrying U2 proteins tagged with the TAP-tag and assembled on site-specifically labeled pre-mRNAs. (A) Schematic representation of site-specifically labeled pre-mRNAs carrying a single ^32^P-labeled phosphate 5’ of the guanosines shown in green. The RNA fragments theoretically remaining after digestion with RNase T1 are indicated by a bar below the sequence. (B) The RNA isolated from the B^act^ complexes carrying the U2 proteins indicated, tagged with the TAP-tag, was analyzed on a denaturing gel with silver-staining of the RNA and autoradiography. The presence of U2, U5L, U5S and U6 snRNA, and the absence of splicing intermediates of the pre-mRNA confirmed B^act^ complex identity. Asterisks indicate the presence of small amount of U1, U4 and ribosomal RNAs, respectively.
(TIF)

S3 Fig Characterization of B^act^, B* and C complexes assembled on site-specifically labeled pre-mRNAs carrying a single ^32^P-labeled phosphate 5’ of the guanosines G452, 460, 467 and 478. (A) Schematic representation of site-specifically labeled pre-mRNAs as described in S2 Fig. (B) Proteins isolated from the B^act^, B* and C complexes, before and after crosslinking, were analyzed by SDS-PAGE. (C). The RNA isolated from these complexes was analyzed by denaturing PAGE and stained with silver. The presence of U2, U5L, U5S and U6 snRNA, and the nearly total absence of splicing intermediates of the pre-mRNA confirmed B^act^ and B* complexes identity. The presence of step 1 products established the identity of the C complex. Species on the gel were quantified using the ImageQuant software. The efficiency of step 1 was determined by the formula: (intron-3’ exon + 5’ exon) / (intron-3’ exon + 5’ exon + pre-mRNA) x 100, and was calculated to be ~ 40%. The asterisk indicates the presence of a small amount of U1 snRNA. (D) Western-blot analysis showing that the transformation from B^act^ to B* was efficient, as revealed by the almost complete dissociation of Cwc24 from the B* complex during catalytic activation [11].
(TIF)

S4 Fig Characterization of B^act^, B* and C complexes assembled on site-specifically labeled pre-mRNAs carrying a single ^32^P-labeled phosphate 5’ of the guanosines G482, 496, 511 and 516. (A) Schematic representation of site-specifically labeled pre-mRNAs as described in S2 Fig. (B) Proteins isolated from the B^act^, B* and C complexes, before and after crosslinking, were analyzed by SDS-PAGE. (C). The RNA isolated from these complexes was analyzed by denaturing PAGE and stained with silver. The presence of U2, U5L, U5S and U6 snRNA, and the nearly total absence of splicing intermediates of the pre-mRNA confirmed B^act^ and B* complexes identity. The presence of step 1 products established the identity of the C complex. Species on the gel were quantified using the ImageQuant software. The efficiency of step 1 was determined by the formula: (intron-3’ exon + 5’ exon) / (intron-3’ exon + 5’ exon + pre-mRNA) x 100, and was calculated to be ~ 40%. The asterisk indicates the presence of a small amount of U1 snRNA. (D) Western-blot analysis showing that the transformation from B^act^ to B* was efficient, as revealed by the almost complete dissociation of Cwc24 from the B* complex during catalytic activation [11].
(TIF)

S5 Fig Identification of proteins crosslinked to site-specifically labeled pre-mRNAs in purified B^act^ complexes. (A–C, upper panels) B^act^ complexes were assembled on the site-specifically labeled pre-mRNA shown using yeast extracts containing proteins–highlighted with colors–tagged with the TAP-tag as indicated or with no tag (‘Untagged’, lanes 1 and 2). Peak fractions of purified B^act^ complexes were UV-irradiated, digested with RNase T1 and then separated on SDS PAGE gels. After transfer to the nitrocellulose membrane, samples were visualized by autoradiography (upper panels) or western blotting, using the PAP antibody complex (lower panels). The addition of the 21 kDa TAP-tag to proteins resulted in an increase in their apparent molecular masses, as shown. (C) Note that UV-irradiation of the B^act^ complex carrying Snu17-TAP led to a shift of Snu17, but also to the disappearance/shift of Pml1 (lane 3), probably owing to their intricate folding [20]. The sizes in kilodaltons of the protein molecular-mass markers are shown to the right of the autoradiography or to the left of the western blot. Asterisks: uncharacterized degradation products of Hsh155.
(TIF)

S6 Fig Quantification of crosslinks. Bands with the highest intensity according to Phosphorimager measurements were designated as having an intensity of 100% and the measurements of other bands were normalized against this value. Error bars represent the standard error of the mean of 2 independent experiments. (A) Quantification of U2 protein crosslinks (related to Fig 3A). (B) Quantification of radioactive spots of proteins crosslinked to the 3’-region-labeled pre-mRNA and separated by 2D gel electrophoresis (related to Figs 3C, 3D and 5A). (C) Quantification of RES complex proteins and Prp8 crosslinks (related to Fig 3A).
(TIF)

S7 Fig Dynamics of protein–pre-mRNA interactions in the region 483–496 of the actin pre-mRNA intron. Schematic representation of site-specifically labeled pre-mRNAs as shown in S2 Fig. Purified B^actΔPrp2^ and reconstituted B* and C complexes were UV-irradiated, digested with RNase T1, and analyzed by SDS-PAGE. Details of the autoradiography of the gel are shown.
(TIF)

S8 Fig An oligoribonucleotide complementary to the 14-nt-long region upstream of the BS inhibits formation of yeast spliceosomes. Wild-type actin pre-mRNA was incubated with a 100-fold molar excess of a 14-nt-long 2’-O-Methyl RNA oligonucleotide complementary to the pre-mRNA sequence nucleotides 447–460 (Anchoring Site Oligo) or with a control oligonucleotide complementary to a sequence of the intron more upstream (nucleotides 415–428), in 20 mM HEPES-KOH pH 7.9. The reaction mixture was incubated at 70°C for 2 min and then cooled to 4°C at 10°C/minute. *In vitro* splicing and B^actΔPrp2^ complex assembly were performed as described in the S1 Text. (Left panel) Glycerol-gradient sedimentation profiles of B^act ΔPrp2^ spliceosomes (formed on body-^32^P-labeled wild-type actin pre-mRNA). 10–30% (v/v) glycerol gradients containing 75 mM KCl were centrifuged for 2 h at 60000 rpm in a TH660 rotor (Sorvall). The radioactivity contained in each fraction was determined by Cherenkov counting and plotted. (Right panel) Glycerol-gradient fractions were digested with Proteinase K and analyzed by denaturing gel electrophoresis and silver staining.
(TIF)

S1 Table Human and yeast SF3a/SF3b proteins. (DOCX)

S2 Table Human and yeast RES complex proteins. (DOCX)

S1 Text Supporting Protocols. (DOCX)
