# Supplementary figures and images for: Dynamic Contacts of U2, RES, Cwc25, Prp8 and Prp45 Proteins with the Pre-mRNA Branch-Site and 3' Splice Site during Catalytic Activation and Step 1 Catalysis in Yeast Spliceosomes
Source: PLoS Genet. 2015 Sep 22;11(9):e1005539. doi: 10.1371/journal.pgen.1005539 (PMC4579134; doi:10.1371/journal.pgen.1005539)

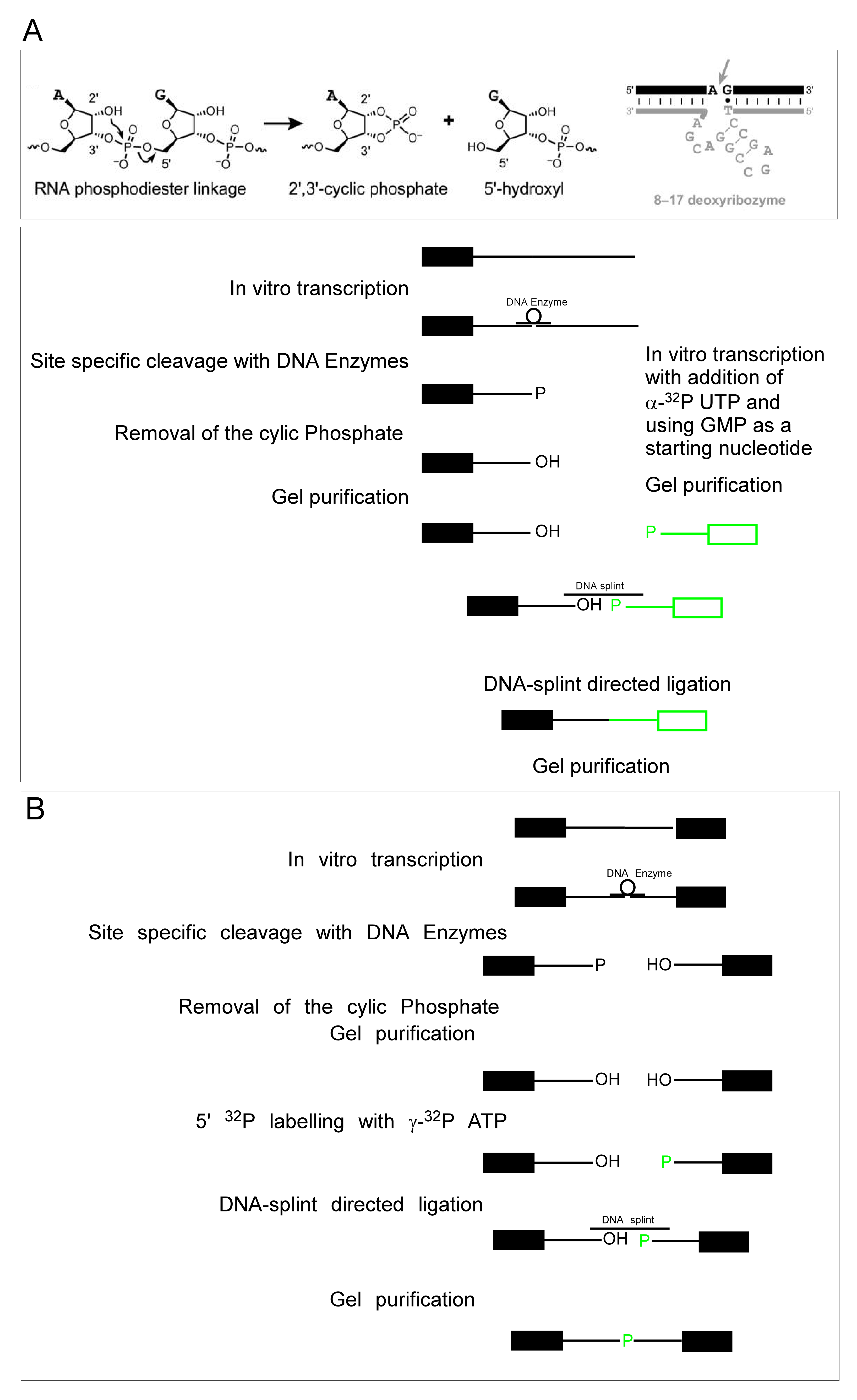

Supplement: S1 Fig — (A) Experimental strategy used for the production of region-specifically labeled pre-mRNA. Upper panels. Left: Reaction mechanism of RNA cleavage by DNA enzymes (adapted from Silverman and Baum [25]). Right: Representation of the 8–17 deoxyribozyme. Base pairing between the target RNA and the recognition arms of the DNA enzymes and the supposed structure and sequence of the catalytic DNA loop are represented schematically (adapted from Silverman and Baum [25]). The pre-mRNA is represented schematically by lines indicating the intron and by rectangles indicating the exons. Radioactively labeled stretches are shown in green. (B) Experimental strategy used for the production of site-specifically labeled pre-mRNA. The pre-mRNA is represented schematically by lines indicating the intron and by rectangles indicating the exons. The 32P introduced by this procedure is shown in green. (TIF) [file pgen.1005539.s001.tif]

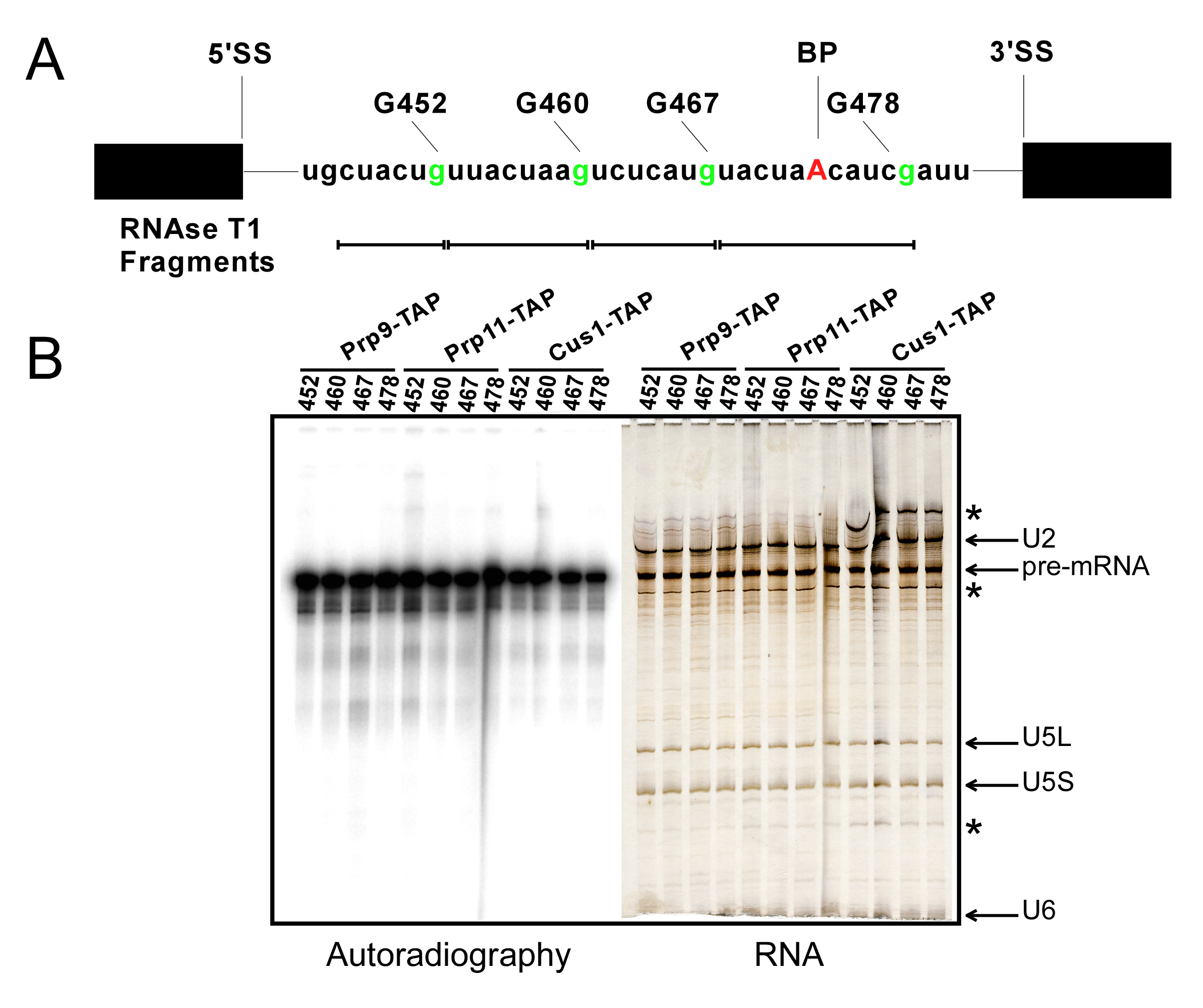

Supplement: S2 Fig — (A) Schematic representation of site-specifically labeled pre-mRNAs carrying a single 32P-labeled phosphate 5’ of the guanosines shown in green. The RNA fragments theoretically remaining after digestion with RNase T1 are indicated by a bar below the sequence. (B) The RNA isolated from the Bact complexes carrying the U2 proteins indicated, tagged with the TAP-tag, was analyzed on a denaturing gel with silver-staining of the RNA and autoradiography. The presence of U2, U5L, U5S and U6 snRNA, and the absence of splicing intermediates of the pre-mRNA confirmed Bact complex identity. Asterisks indicate the presence of small amount of U1, U4 and ribosomal RNAs, respectively. (TIF) [file pgen.1005539.s002.tif]

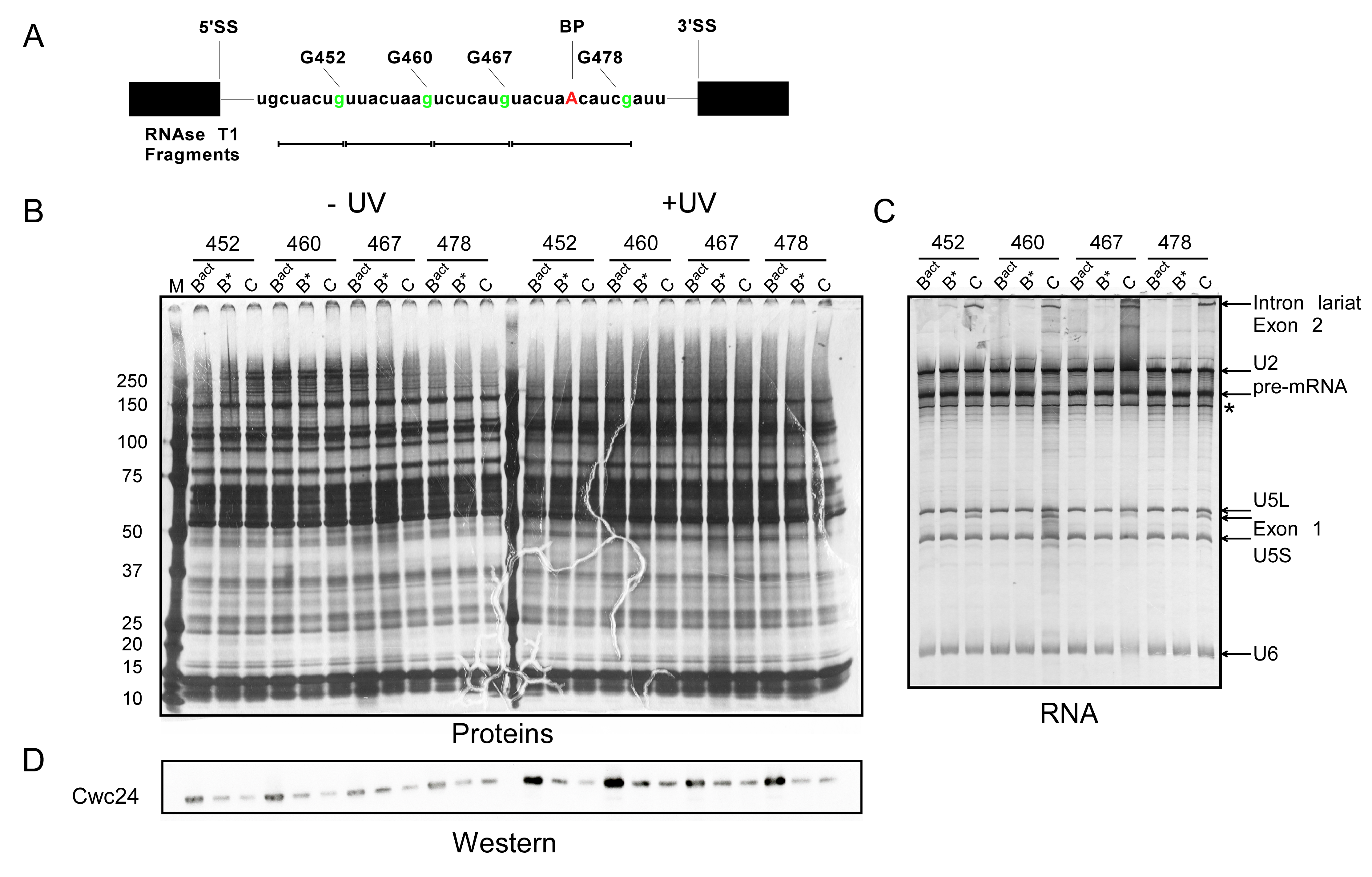

Supplement: S3 Fig — (A) Schematic representation of site-specifically labeled pre-mRNAs as described in S2 Fig. (B) Proteins isolated from the Bact, B* and C complexes, before and after crosslinking, were analyzed by SDS-PAGE. (C). The RNA isolated from these complexes was analyzed by denaturing PAGE and stained with silver. The presence of U2, U5L, U5S and U6 snRNA, and the nearly total absence of splicing intermediates of the pre-mRNA confirmed Bact and B* complexes identity. The presence of step 1 products established the identity of the C complex. Species on the gel were quantified using the ImageQuant software. The efficiency of step 1 was determined by the formula: (intron-3’ exon + 5’ exon) / (intron-3’ exon + 5’ exon + pre-mRNA) x 100, and was calculated to be ~ 40%. The asterisk indicates the presence of a small amount of U1 snRNA. (D) Western-blot analysis showing that the transformation from Bact to B* was efficient, as revealed by the almost complete dissociation of Cwc24 from the B* complex during catalytic activation [11]. (TIF) [file pgen.1005539.s003.tif]

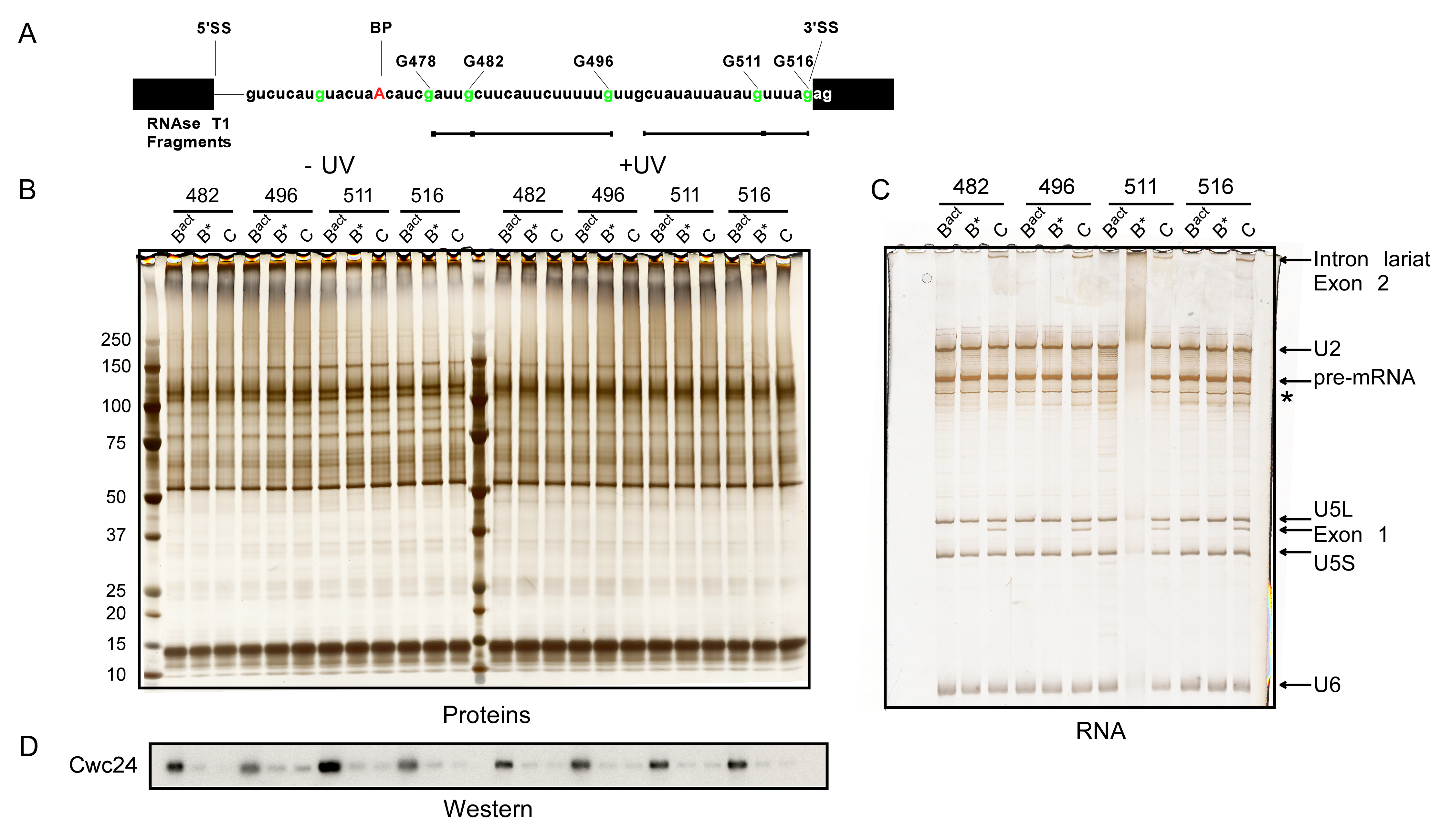

Supplement: S4 Fig — (A) Schematic representation of site-specifically labeled pre-mRNAs as described in S2 Fig. (B) Proteins isolated from the Bact, B* and C complexes, before and after crosslinking, were analyzed by SDS-PAGE. (C). The RNA isolated from these complexes was analyzed by denaturing PAGE and stained with silver. The presence of U2, U5L, U5S and U6 snRNA, and the nearly total absence of splicing intermediates of the pre-mRNA confirmed Bact and B* complexes identity. The presence of step 1 products established the identity of the C complex. Species on the gel were quantified using the ImageQuant software. The efficiency of step 1 was determined by the formula: (intron-3’ exon + 5’ exon) / (intron-3’ exon + 5’ exon + pre-mRNA) x 100, and was calculated to be ~ 40%. The asterisk indicates the presence of a small amount of U1 snRNA. (D) Western-blot analysis showing that the transformation from Bact to B* was efficient, as revealed by the almost complete dissociation of Cwc24 from the B* complex during catalytic activation [11]. (TIF) [file pgen.1005539.s004.tif]

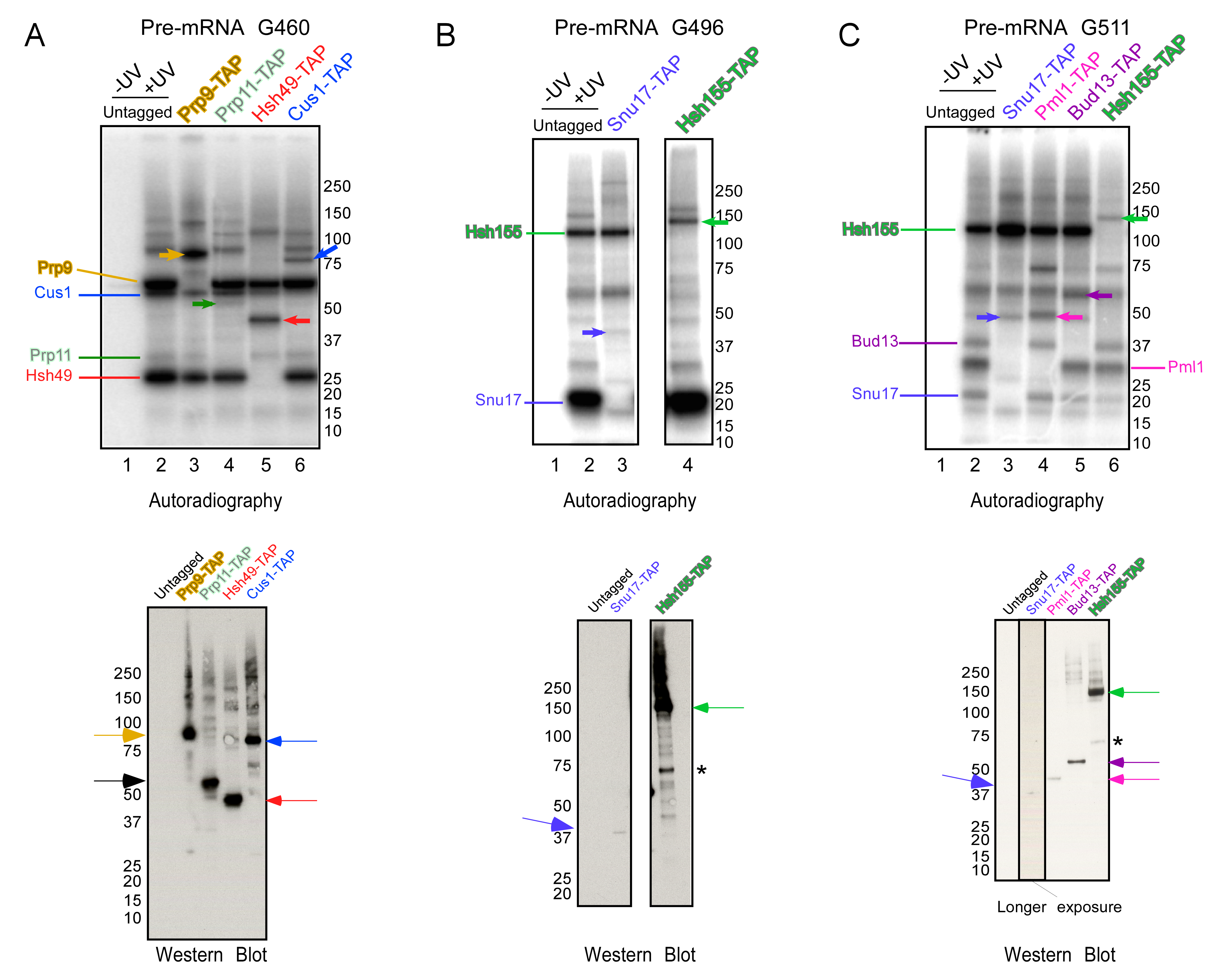

Supplement: S5 Fig — (A–C, upper panels) Bact complexes were assembled on the site-specifically labeled pre-mRNA shown using yeast extracts containing proteins–highlighted with colors–tagged with the TAP-tag as indicated or with no tag (‘Untagged’, lanes 1 and 2). Peak fractions of purified Bact complexes were UV-irradiated, digested with RNase T1 and then separated on SDS PAGE gels. After transfer to the nitrocellulose membrane, samples were visualized by autoradiography (upper panels) or western blotting, using the PAP antibody complex (lower panels). The addition of the 21 kDa TAP-tag to proteins resulted in an increase in their apparent molecular masses, as shown. (C) Note that UV-irradiation of the Bact complex carrying Snu17-TAP led to a shift of Snu17, but also to the disappearance/shift of Pml1 (lane 3), probably owing to their intricate folding [20]. The sizes in kilodaltons of the protein molecular-mass markers are shown to the right of the autoradiography or to the left of the western blot. Asterisks: uncharacterized degradation products of Hsh155. (TIF) [file pgen.1005539.s005.tif]

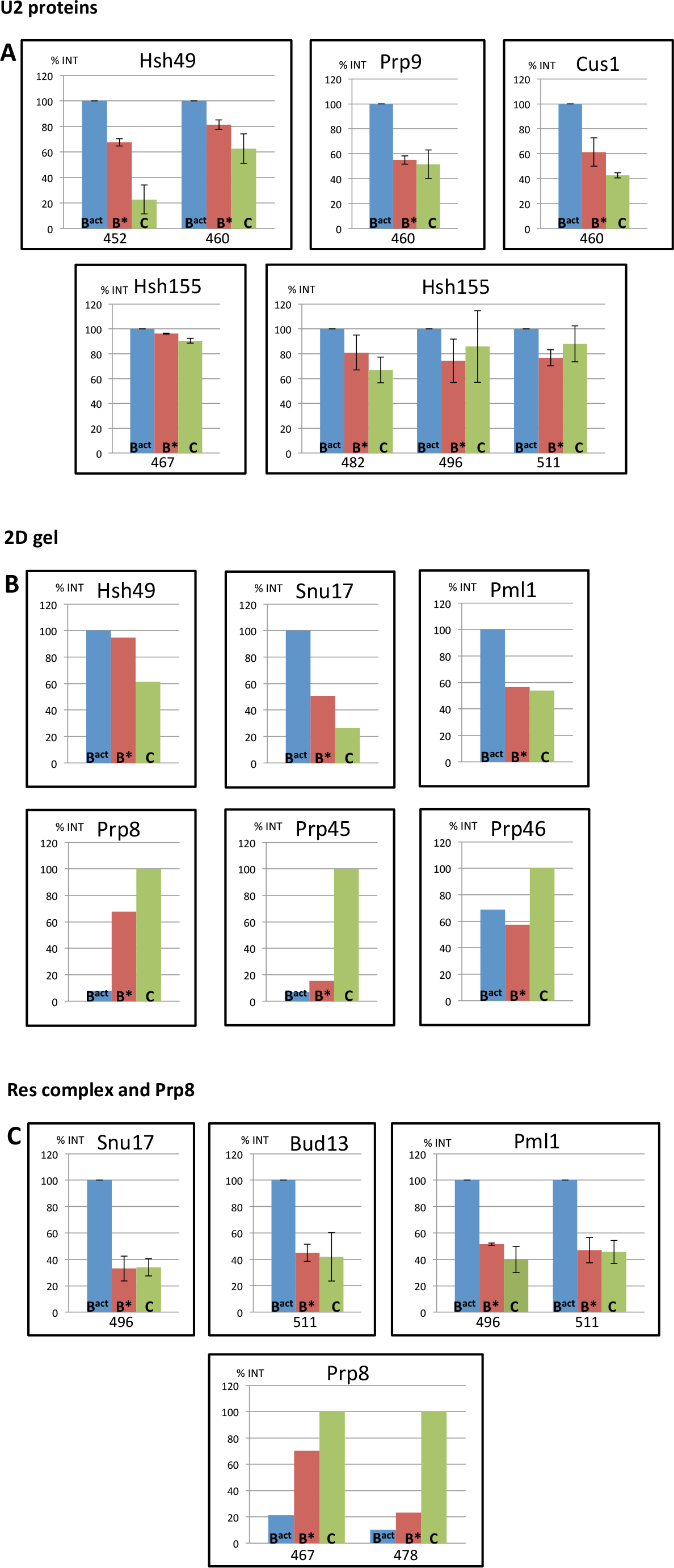

Supplement: S6 Fig — Bands with the highest intensity according to Phosphorimager measurements were designated as having an intensity of 100% and the measurements of other bands were normalized against this value. Error bars represent the standard error of the mean of 2 independent experiments. (A) Quantification of U2 protein crosslinks (related to Fig 3A). (B) Quantification of radioactive spots of proteins crosslinked to the 3’-region-labeled pre-mRNA and separated by 2D gel electrophoresis (related to Figs 3C, 3D and 5A). (C) Quantification of RES complex proteins and Prp8 crosslinks (related to Fig 3A). (TIF) [file pgen.1005539.s006.tif]

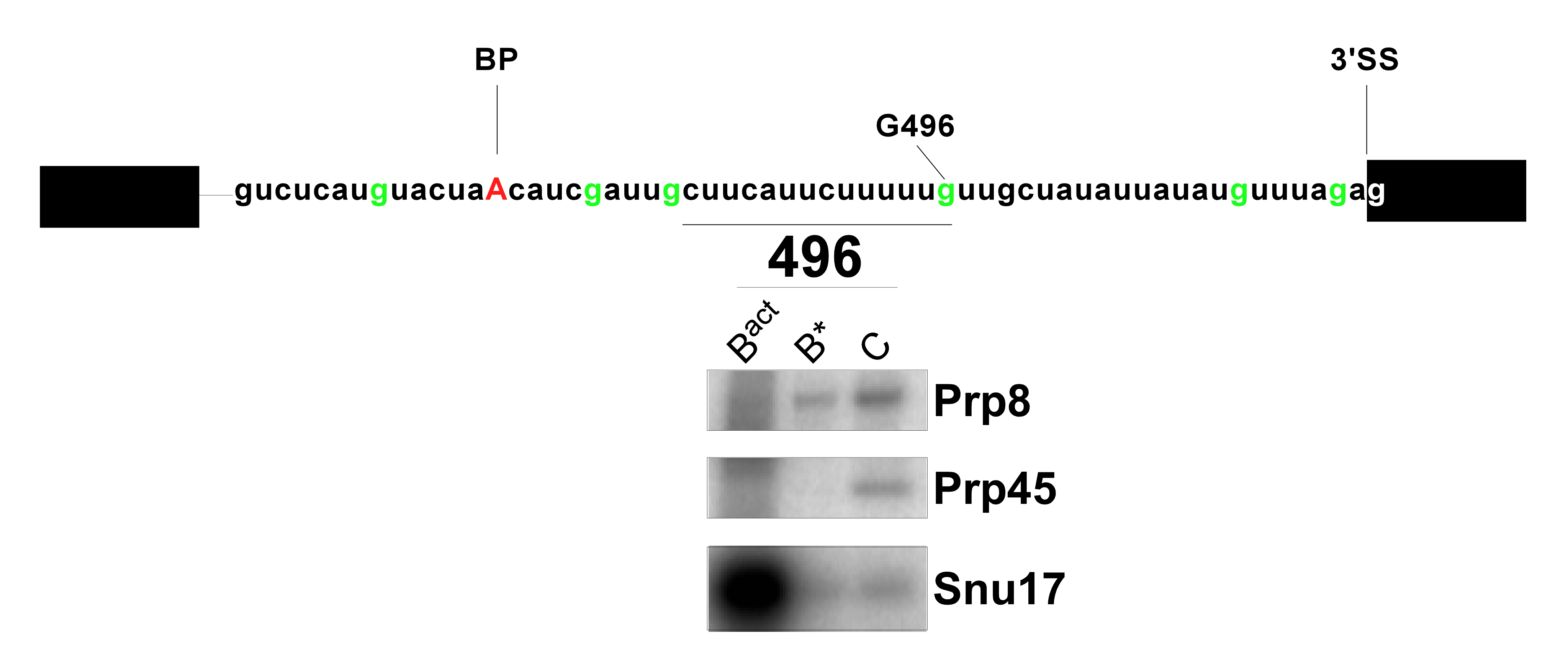

Supplement: S7 Fig — Schematic representation of site-specifically labeled pre-mRNAs as shown in S2 Fig. Purified BactΔPrp2 and reconstituted B* and C complexes were UV-irradiated, digested with RNase T1, and analyzed by SDS-PAGE. Details of the autoradiography of the gel are shown. (TIF) [file pgen.1005539.s007.tif]

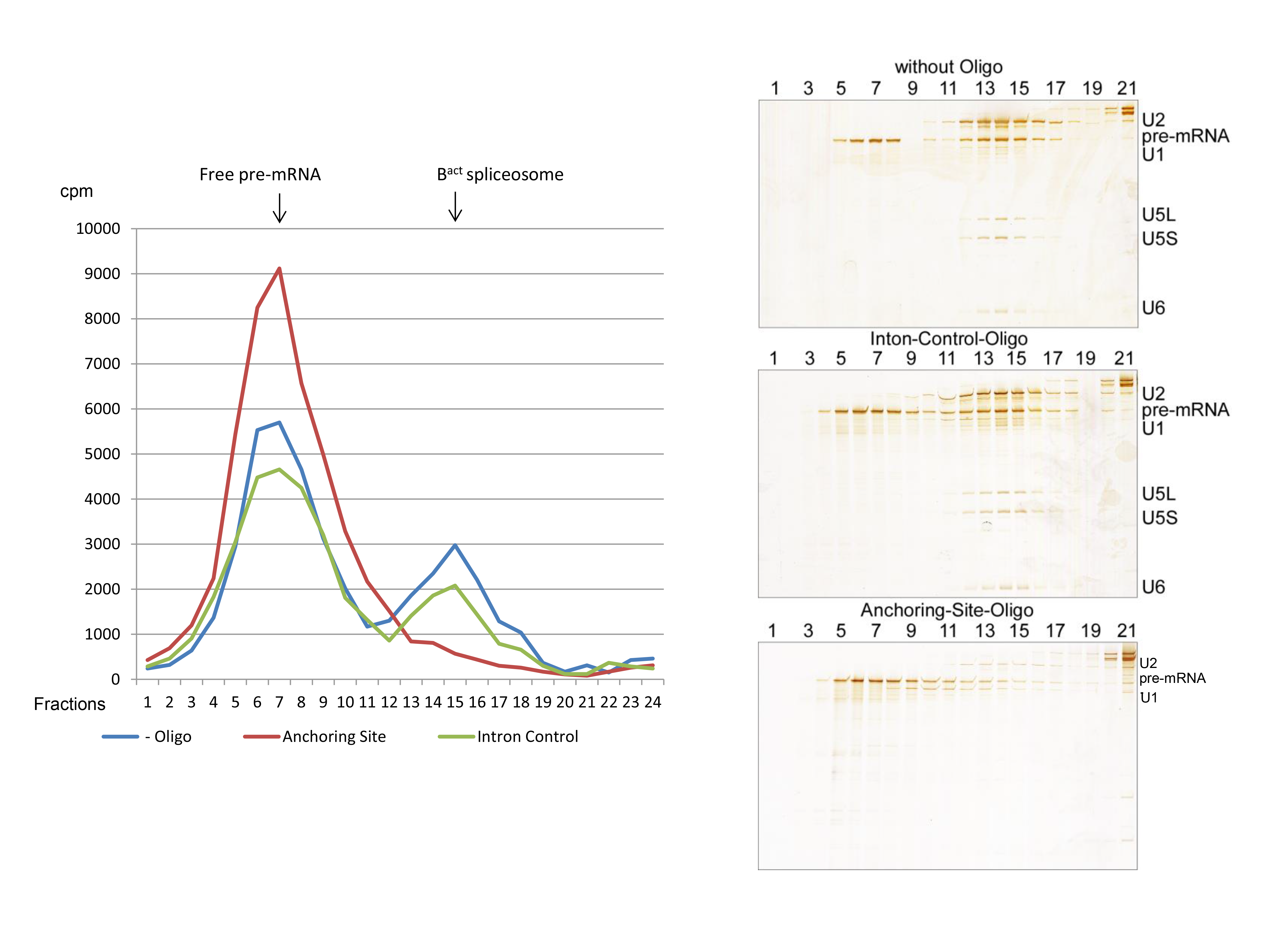

Supplement: S8 Fig — Wild-type actin pre-mRNA was incubated with a 100-fold molar excess of a 14-nt-long 2’-O-Methyl RNA oligonucleotide complementary to the pre-mRNA sequence nucleotides 447–460 (Anchoring Site Oligo) or with a control oligonucleotide complementary to a sequence of the intron more upstream (nucleotides 415–428), in 20 mM HEPES-KOH pH 7.9. The reaction mixture was incubated at 70°C for 2 min and then cooled to 4°C at 10°C/minute. In vitro splicing and BactΔPrp2 complex assembly were performed as described in the S1 Text. (Left panel) Glycerol-gradient sedimentation profiles of Bact ΔPrp2 spliceosomes (formed on body-32P-labeled wild-type actin pre-mRNA). 10–30% (v/v) glycerol gradients containing 75 mM KCl were centrifuged for 2 h at 60000 rpm in a TH660 rotor (Sorvall). The radioactivity contained in each fraction was determined by Cherenkov counting and plotted. (Right panel) Glycerol-gradient fractions were digested with Proteinase K and analyzed by denaturing gel electrophoresis and silver staining. (TIF) [file pgen.1005539.s008.tif]
