# Supplementary material for: Dynamic Contacts of U2, RES, Cwc25, Prp8 and Prp45 Proteins with the Pre-mRNA Branch-Site and 3' Splice Site during Catalytic Activation and Step 1 Catalysis in Yeast Spliceosomes
Source: PLoS Genet. 2015 Sep 22;11(9):e1005539. doi: 10.1371/journal.pgen.1005539 (PMC4579134; doi:10.1371/journal.pgen.1005539)
Supplement: S1 Table — (DOCX) [file pgen.1005539.s009.docx]

**S1 Table Human and yeast SF3a/SF3b proteins**

| **Protein**  **Name** | **Gene**  **Name** | **MW**  **(kDa)** | **MW+ tag**  **(kDa)** | **Human Protein**  **Name** | **Region of Site-specific**  **UV-crosslinking (This work)** |
| --- | --- | --- | --- | --- | --- |
| Rse1 | YML049C | 153.8 | - | [SF3b130](file:///\\fs10700.bpc.mpg.de\Experiments%2006:07:08\Experiments%202006\splicing_database_MAC_MAY20\gene_names.xls#A5:O5) (SAP130) | ND |
| Hsh155 | YMR288W | 110 | 131 | [SF3b155](file:///\\fs10700.bpc.mpg.de\Experiments%2006:07:08\Experiments%202006\splicing_database_MAC_MAY20\gene_names.xls#A3:O3) (SAP155) | 461–467 and  479–482, 483–496, 500–511 |
| Prp9 | YDL030W | 63 | 84 | [SF3a60](file:///\\fs10700.bpc.mpg.de\Experiments%2006:07:08\Experiments%202006\splicing_database_MAC_MAY20\gene_names.xls#A10:O10) (SAP61) | 453–460 |
| Cus1 | YMR240C | 50.2 | 71.2 | [SF3b145](file:///\\fs10700.bpc.mpg.de\Experiments%2006:07:08\Experiments%202006\splicing_database_MAC_MAY20\gene_names.xls#A4:O4) (SAP145) | 453–460 |
| Prp21 | YJL203W | 33 | - | [SF3a120](file:///\\fs10700.bpc.mpg.de\Experiments%2006:07:08\Experiments%202006\splicing_database_MAC_MAY20\gene_names.xls#A12:O12) (SAP114) | ND |
| Prp11 | YDL043C | 29.9 | 50.9 | [SF3a66](file:///\\fs10700.bpc.mpg.de\Experiments%2006:07:08\Experiments%202006\splicing_database_MAC_MAY20\gene_names.xls#A11:O11) (SAP62) | 447–452, 453–460 |
| Lea1 | YPL213W | 27.2 | - | [U2-A'](file:///\\fs10700.bpc.mpg.de\Experiments%2006:07:08\Experiments%202006\splicing_database_MAC_MAY20\gene_names.xls#A45:O45) | ND |
| Hsh49 | YOR319W | 24.5 | 45.5 | [SF3b49](file:///\\fs10700.bpc.mpg.de\Experiments%2006:07:08\Experiments%202006\splicing_database_MAC_MAY20\gene_names.xls#A6:O6) (SAP49) | 447–452, 453–460 |
| Msl1 | YIR009W | 12.8 | - | [U2-B''](file:///\\fs10700.bpc.mpg.de\Experiments%2006:07:08\Experiments%202006\splicing_database_MAC_MAY20\gene_names.xls#A44:O44) | ND |
| - | - | - | - | SF3b14a | - |
| Rds3 | YPR094W | 12.3 | 33.3 | [SF3b14b](file:///\\fs10700.bpc.mpg.de\Experiments%2006:07:08\Experiments%202006\splicing_database_MAC_MAY20\gene_names.xls#A7:O7) | - |
| Ysf3 | YNL138W-A | 10 | 31 | [SF3b10](file:///\\fs10700.bpc.mpg.de\Experiments%2006:07:08\Experiments%202006\splicing_database_MAC_MAY20\gene_names.xls#A8:O8) | - |

SF3b (Rse1, Hsh155, Cus1, Hsh49, Rds3, Ysf3), SF3a (Prp9, 11, 21). ND = Not determined.

Human U2 proteins could be cross-linked to a 20-nucleotides-long region of the pre-mRNA upstream of the branch-site (the so-called “anchoring site”), in the order SF3b49 - SF3a120 - SF3a60 - SF3b145 - SF3a66 - SF3b155 [1,2]

1. Champion-Arnaud P, Reed R The prespliceosome components SAP 49 and SAP 145 interact in a complex implicated in tethering U2 snRNP to the branch site. Genes Dev. 1994;8: 1974-1983.

2. Gozani O, Feld R, Reed R Evidence that sequence-independent binding of highly conserved U2 snRNP proteins upstream of the branch site is required for assembly of spliceosomal complex A. Genes Dev. 1996;10: 233-243.
