# Supplementary material for: Dynamic Contacts of U2, RES, Cwc25, Prp8 and Prp45 Proteins with the Pre-mRNA Branch-Site and 3' Splice Site during Catalytic Activation and Step 1 Catalysis in Yeast Spliceosomes
Source: PLoS Genet. 2015 Sep 22;11(9):e1005539. doi: 10.1371/journal.pgen.1005539 (PMC4579134; doi:10.1371/journal.pgen.1005539)
Supplement: S2 Table — (DOCX) [file pgen.1005539.s010.docx]

**S2 Table Human and yeast RES complex proteins**

| **Protein**  **Name** | **Gene**  **Name** | **MW**  **(kDa)** | **MW+ tag**  **(kDa)** | **Human Protein**  **Name** | **Region of Site-specific**  **UV-crosslinking (This work)** |
| --- | --- | --- | --- | --- | --- |
| Bud13 | YGL174W | 30.5 | 51.5 | MGC13125 | 500–511 |
| Pml1 | YLR016C | 23.6 | 44.6 | SNIP1? | 483–496, 500–511 |
| Ist3/Snu17 | YIR005W | 17 | 48 | CGI-79 | 483–496 |
