# Supplementary material for: Dynamic Contacts of U2, RES, Cwc25, Prp8 and Prp45 Proteins with the Pre-mRNA Branch-Site and 3' Splice Site during Catalytic Activation and Step 1 Catalysis in Yeast Spliceosomes
Source: PLoS Genet. 2015 Sep 22;11(9):e1005539. doi: 10.1371/journal.pgen.1005539 (PMC4579134; doi:10.1371/journal.pgen.1005539)
Supplement: S1 Text — (DOCX) [file pgen.1005539.s011.docx]

**Dynamic contacts of U2, RES, Cwc25, Prp8 and Prp45 proteins with the pre-mRNA branch-site and 3' splice site during catalytic activation and step 1 catalysis in yeast spliceosomes**

Cornelius Schneider, Dmitry E. Agafonov, Jana Schmitzová, Klaus Hartmuth, Patrizia Fabrizio^1^ and Reinhard Lührmann^1^

Max Planck Institute for Biophysical Chemistry, Göttingen, Germany

Department of Cellular Biochemistry

^1^ Corresponding authors

**Supporting Protocols:**

- Splicing substrates
- Production of site‑ and region‑specific labeled pre‑mRNAs
- Preparation of yeast whole‑cell extract
- Purification of spliceosomes
- *In vitro* splicing reconstitution
- UV crosslinking of spliceosomal complexes
- Immunoprecipitation of proteins from crosslinked B^act^ ^ΔPrp2^ spliceosomal complexes
- Cwc25 truncation mutants and protein purification
- Western blotting
- 2D gel electrophoresis and mass spectrometry
- PCR primers
- DNA enzymes
- DNA splint oligonucleotides
- 2’-O-Methyl RNA oligonucleotides

*Splicing substrates*

Yeast actin pre‑mRNAs used for assembly of spliceosomal complexes were generated by run‑off transcription *in vitro* with T7 RNA polymerase. All transcripts were tagged with three MS2 stem-loops at the 5’ end [1] and were 687 nucleotides long. For transcription *in vitro* with GMP as a starting nucleotide, 1 mM GMP and 0.1 mM GTP were added to the reaction. All transcription reactions were incubated at 37 °C for 3 hrs. After DNA template digestion with RQ1 DNase at 37 °C for 15 min, transcripts were purified by electrophoresis through 5% polyacrylamide gels containing 8 M urea. The transcription product was visualized either by UV shadowing at 312 nm for non‑radioactive transcriptions or by autoradiography for radioactive transcriptions. Transcripts were excised from the gel and eluted passively overnight at room temperature in 20 mM TRIS‑HCl pH 7.5, 150 mM NaCl, 0.2 mM EDTA pH 8.0, 0.5% (w/v) SDS.

*Production of site‑ and region‑specific labeled pre‑mRNAs*

Site‑ and region‑specific labeled pre‑mRNAs were produced by ligation of RNA fragments prepared previously by site‑specific cleavage of the pre‑mRNA with DNA enzymes essentially as described previously [2,3]. RNA fragments were ligated by DNA splint‑directed RNA ligation essentially as described previously [4].

*Preparation of yeast whole‑cell extract*

The yeast strain *prp2*‑1 [strain 3.2.AID; alpha, *prp2*‑*1*, *ade2*, *his3*, *lys2*‑*801*, *ura3*, carrying a G360D mutation in Prp2; [5]] kindly provided by Ren‑Jang Lin, was grown to OD_600_ = 3–5. The cells were harvested and washed with ice‑cold water. Each gram of washed and pelleted cells were suspended in 0.7 ml AGK buffer (20 mM HEPES‑KOH pH 7.9, 400 mM KCl, 1.5 mM MgCl_2_, 8% (v/v) glycerol) containing 1 mM DTT and protease inhibitors (Roche) and drops of the mixture were frozen in liquid nitrogen. Frozen yeast‑cell beads were ground at 18000 rpm in an ultracentrifugal mill (ZM 200, Retsch), and the resulting powder was thawed in a water bath at room temperature. The crude lysate was centrifuged twice at 4 °C in a SS34 rotor (Sorvall) at 17000 rpm for 30 min. The supernatant was then centrifuged for 1 h in a T647.5 rotor (Thermo Scientific) at 42000 rpm. The resulting cleared‑lysate (middle phase, ca. 70% of total volume) was dialyzed twice for 1 h 30 min, each time with dialysis membranes (Spectrum, membrane cut‑off 6–8 kDa) against 5 L of buffer D (20 mM HEPES‑KOH pH 7.9, 50 mM KCl, 0.2 mM EDTA, 20% (v/v) glycerol, 0.5 mM DTT, 0.5 mM PMSF, 2 mM benzamidine). The dialyzed extract was centrifuged in a SS34 rotor (Sorvall) for 10 min at 4°C, divided into aliquots, frozen in liquid nitrogen, and stored at –80 °C.

*Purification of spliceosomes*

Preparative purifications of the B^act^ ^ΔPrp2^ spliceosome were performed as described previously [1,6]. Briefly, *in vitro* splicing reactions with 2 nM pre‑mRNA (containing ^32^P‑labeled pre‑mRNA, ca. 20–100 cpm/fmole) and 40% (v/v) yeast whole-cell extract from the *prp2*‑1 strain heat-treated at 35 ^o^C for 30 min, were incubated for 30–40 min at 23^o^C. For some experiments protocol 1 was used; the reaction mixtures were layered on 10–30% (v/v) glycerol gradients (20 mM Hepes‑KOH pH 7.9, 150 mM KCl, 1.5 mM MgCl_2_, 0.01% NP40) and centrifuged in a SureSpin rotor (Kendro) at 23000 rpm at 4 ^o^C for 14 h. Gradients were fractionated manually into 24 fractions from top to bottom. Spliceosomes in fractions 15–20 were pooled and affinity-selected on 0.6 ml amylose resin (NEB) in 10 ml columns (BioRad). For the majority of the experiments protocol 2 was used instead; the first glycerol gradient was omitted and the splicing reaction was first clarified by centrifugation in a Fiberlite rotor F14‑14x50cy (Thermo) for 10 min at 9000 rpm (13300 g) and then affinity-selected directly on amylose resin. The matrix‑bound complexes were washed with 20 ml GK150 and 10 ml GK75 buffer (150 or 75 mM KCl, 20 mM Hepes‑KOH pH 7.9, 1.5 mM MgCl_2_, 5% v/v glycerol, 0.01% NP40). Spliceosomes were eluted with 0.6 ml of GK75 containing 12 mM maltose. Eluted complexes were used directly for reconstitution experiments and then subjected to a 10–30% glycerol gradient in GK75 buffer which was centrifuged at 60000 rpm for 2 hrs at 4 °C. The amounts and molarity of the eluted spliceosomes was estimated based on the specific activity of the pre‑mRNA and the radioactivity determined by Cherenkov counting.

*Splicing reconstitution* in vitro

Reconstitution *in vitro* was performed essentially as described previously [6]. The reconstitution reaction was performed in GK75 buffer, in the presence of 2 U/µl RNAsin, a 10 fold molar excess of recombinant protein over purified B^actΔPrp2^ spliceosomes, 2 mM ATP and 2.5 mM MgCl_2_. For reconstitution of B* complexes, Prp2 and Spp2 recombinant proteins were added to the reaction; for reconstitution of C complexes, recombinant Cwc25 was also added. The reaction was then incubated at 23 °C for 40 min. Reconstitution efficiency was determined by western blot analysis to examine the release of Cwc24. For same experiments reconstitution efficiency was determined by analytic 10–30% glycerol gradient (in GK75 buffer) [6] for the B^act^ to B* transition or by pre‑mRNA analysis on a denaturing polyacrylamide gel for the B* to C transition.

*UV crosslinking of spliceosomal complexes*

Approximately 0.1–2 pmol of purified B^act^ ^ΔPrp2^ spliceosomes were pipetted in a thin layer onto a pre*‑*cooled metal block covered with Parafilm and then irradiated for 30 sec with UV light at 254 nm on ice, essentially as described previously [7]. To the irradiated and non‑irradiated control samples, SDS and EDTA were added to final concentrations of 0.1% and 10 mM, respectively, and spliceosomes were incubated for 10 min at 70 °C. The mixture was then allowed to cool to 37 °C, and 1 μl RNase T1 (1000 units/μl Ambion) was added: Incubation was continued for 30 min at 37 °C followed by 30 min at 55 °C. After addition of 0.3 M NaOAc, 1 μl of Glycoblue and 4 volumes of ethanol, the proteins crosslinked to ^32^P‑labeled RNA fragments were precipitated overnight at –20 °C. After centrifugation at 13000 rpm for 30 min the pellet was washed once with 70% ethanol and re‑suspended in NuPAGE loading dye for analysis by Novex NuPAGE gels (Invitrogen) electrophoresis.

*Immunoprecipitation of proteins from crosslinked B^act^ ^ΔPrp2^ spliceosomal complexes*

Yeast strains carrying the *prp2*‑*1* mutation [5] and C‑terminally TAP tagged proteins of interest were created as described previously [8] using plasmid pBS1539-Psc [9]. In these extracts, yeast B^act^ ^ΔPrp2^ spliceosomal complexes were assembled as described above [6] on site‑specifically ^32^P‑labeled pre‑mRNA [2,4] and then purified. One pmol of spliceosomes was eluted in 400 µl GK75 (75 mM KCl, 20 mM HEPES‑KOH, pH 7.9, 1.5 mM MgCl_2_, 0.01% NP40, 5% glycerol) and subjected to UV crosslinking at 254 nm for 30 sec. The complexes were first precipitated with ethanol and then denatured by incubation with 3% SDS in NET buffer (150 mM NaCl, 50 mM Tris‑HCl pH 7.5) at 70 °C for 10 min. After diluting 1:20 with NET buffer, the RNA was digested with a final concentration of 6 units/µl RNase T1 (Ambion) at 37 °C for 1 h, essentially as described previously [10]. TAP-tagged proteins were immunoprecipitated directly from the digestion reaction. Proteins were eluted from the IgG beads (GE Healthcare) by adding 1 x NuPAGE LDS sample buffer (Invitrogen) and incubating at 70 °C for 10 min, and separated on Novex NuPAGE gels (Invitrogen). Western blotting was probed using the PAP complex antibody (peroxidase‑anti‑peroxidase complex, Sigma). After transfer, but before probing of the proteins, the membrane was subjected to autoradiography.

*Cwc25 truncation mutants and protein purification*

Recombinant proteins were full‑length Prp2, Cwc25 and truncated Spp2 from which amino-acid residues 1–35 had been removed [6]. All proteins were fused to a hexahistidine tag (for Prp2 and Spp2, at the C terminus, in plasmid pET21a; for Cwc25, at the N terminus, in plasmid pETM11) for affinity purification. The truncation mutants 1–102, 1–125 and 1–168 of Cwc25 were obtained by PCR and cloned into the NcoI site of pETM11. All mutants were expressed in Rosetta II *E. coli* cells. Cells were lysed in buffer containing 50 mM HEPES pH7.5, 1 M NaCl, 15% glycerol, 10 mM imidazole and 2 mM β‑mercaptoethanol. The lysates were washed with 2 M LiCl, then with 5% and 10% buffer B and eluted with 50% buffer B (50 mM HEPES pH 7.5, 300 mM NaCl, 10% Glycerol, 250 mM imidazole, 2 mM β−mercaptoethanol). After Ni-NTA chromatography, chromatography on a Superdex S75 (16/600) was used. The column was equilibrated with a buffer containing 20 mM HEPES pH 7.5, 200 mM NaCl, 5% glycerol and 2 mM DTT. The N-terminal histidine tag was cleaved off overnight with TEV protease in a ratio 1:100.

*Western blotting*

Proteins were obtained from affinity‑purified spliceosomes. Proteins separated by SDS‑PAGE were transferred to a nitrocellulose membrane (Protan BA83 nitrocellulose, 0.2 µm; Schleicher & Schuell) by a wet-blot procedure. Probing was performed with rabbit polyclonal antibodies against Cwc24 (gift from Kum‑Loong Boon). Secondary goat anti‑rabbit antibodies (Jackson Immunoresearch, USA) were used at 1‑to‑50000 dilution. PAP complex was added in a 1‑to‑3000 dilution and incubated for 1 h at room temperature. The blots were developed using an ECL kit (GE Healthcare) and exposed to film (Kodak).

*2D gel electrophoresis and mass spectrometry*

Two-dimensional gel-electrophoresis of affinity-purified spliceosomal complexes was performed as described in [11] and proteins were stained with Ruthenium (II) tris (bathophenantroline disulfonate) RuBPS (RubiLAB) an analog of the Sypro Ruby dye [12]. In gel fluorescence was detected by Fujifilm FLA-7000 scanner. For mass spectrometry, Coomassie or silver-stained protein-spots were cut out of the 2D gel and proteins were digested in-gel with trypsin and extracted. The extracted peptides were analyzed in a liquid-chromatography coupled electrospray ionization quadropole time of flight mass spectrometer (LTQ Orbitrap XL) under standard conditions. Proteins were identified by searching fragment spectra against the NCBI non-redundant (nr) database using Mascot as a search engine.

*PCR primers for amplification of the 3’ region of actin DNA*

| 426→fwd |  |
| --- | --- |

TAATACGACTCACTATAGTGATAT TCTTCTTTTATTTGC

Actin rev

GGAATTCCCCTTCATCACCAA

*DNA enzymes*

A8-17-426

AAAAGAAGAATATCATCCGAGCCGGACGAATCACTTATCACGA

A8-17-460

GTTAGTACATGAGATCCGAGCCGGACGATAGTAACAGTAGC

E1111-452

CATGAGACTTAGTAATGTCAGCGACACGAAGTAGCAAATAAAAG

E1111-467

AATCGATGTTAGTATGTCAGCGACACGAATGAGACTTAGTAAC

E1111-478

AAAGAATGAAGCAATTGTCAGCGACACGAAATGTTAGTACATGAG

E1111-482

ACAAAAAGAATGAAGTGTCAGCGACACGAAATCGATGTTAGTACA

E1111-496

ACATATAATATAGCAATGTCAGCGACACGAAAAAAGAATGAAGCA

E1111-511

AGCAACCTCTAAATGTCAGCGACACGAATATAATATAGCAACA

*DNA splint oligonucleotides*

Splint 426

ATAAAAGAAGAATATCACTATCACTTATCACGAAA

Splint 452-478

GTGATATTCTTCTTTTATTTGCTACTGTTACTAAGTCTCATGTACTAACATCGATTGCTTCATTCTTTTTGTTG

Splint 496-511

CCAAAGCAGCAACCTCTAAACATATAATATAGCAACAAAAAGAATGAAGCAATCGATGTTAGTACATGAGAC

*2’-O-Methyl RNA oligonucleotides:*

Anchoring site: CUUAGUAACAGUAG

Intron control: CACUAUCACUUAUC

**Supporting References**

1. Fabrizio P, Dannenberg J, Dube P, Kastner B, Stark H, Urlaub H, et al. The evolutionarily conserved core design of the catalytic activation step of the yeast spliceosome. Mol Cell. 2009;36: 593-608.

2. Silverman SK, Baum DA Use of deoxyribozymes in RNA research. Methods Enzymol. 2009;469: 95-117.

3. Cameron V, Uhlenbeck OC 3'-Phosphatase activity in T4 polynucleotide kinase. Biochemistry. 1977;16: 5120-5126.

4. Moore MJ, Sharp PA Site-specific modification of pre-mRNA: the 2'-hydroxyl groups at the splice sites. Science. 1992;256: 992-997.

5. Yean SL, Lin RJ U4 small nuclear RNA dissociates from a yeast spliceosome and does not participate in the subsequent splicing reaction. Mol Cell Biol. 1991;11: 5571-5577.

6. Warkocki Z, Odenwälder P, Schmitzová J, Platzmann F, Stark H, Urlaub H, et al. Reconstitution of both steps of Saccharomyces cerevisiae splicing with purified spliceosomal components. Nat Struct Mol Biol. 2009;16: 1237-1243.

7. Urlaub H, Hartmuth K, Lührmann R A two-tracked approach to analyze RNA-protein crosslinking sites in native, nonlabeled small nuclear ribonucleoprotein particles. Methods. 2002;26: 170-181.

8. Puig O, Caspary F, Rigaut G, Rutz B, Bouveret E, Bragado-Nilsson E, et al. The tandem affinity purification (TAP) method: a general procedure of protein complex purification. Methods. 2001;24: 218-229.

9. Kramer K, Sachsenberg T, Beckmann BM, Qamar S, Boon KL, Hentze MW, et al. Photo-cross-linking and high-resolution mass spectrometry for assignment of RNA-binding sites in RNA-binding proteins. Nat Methods. 2014;11: 1064-1070.

10. Urlaub H, Hartmuth K, Kostka S, Grelle G, Luhrmann R A general approach for identification of RNA-protein cross-linking sites within native human spliceosomal small nuclear ribonucleoproteins (snRNPs). Analysis of RNA-protein contacts in native U1 and U4/U6.U5 snRNPs. J Biol Chem. 2000;275: 41458-41468.

11. Agafonov DE, Deckert J, Wolf E, Odenwälder P, Bessonov S, Will CL, et al. Semiquantitative Proteomic Analysis of the Human Spliceosome via a Novel Two-Dimensional Gel Electrophoresis Method. Mol Cell Biol. 2011;31: 2667-2682.

12. Rabilloud T, Strub JM, Luche S, van Dorsselaer A, Lunardi J A comparison between Sypro Ruby and ruthenium II tris (bathophenanthroline disulfonate) as fluorescent stains for protein detection in gels. Proteomics. 2001;1: 699-704.
